# Cellulose Ionogels: Unraveling Structure–Property Relationships Through Multiscale In-Situ Characterization and Theoretical Modeling

**DOI:** 10.3390/gels12090837

**Published:** 2026-09-12

**Authors:** Jia Wei, Ziyan He, Jingtao Ruan, Junjie Ou, Wen Zhang, Bin Tan, Xiaoheng He, Zhen Wang, Yufei Tang

**Affiliations:** 1 State Key Laboratory of Water Engineering Ecology and Environment in Arid Area, School of Eco-Environmental and Chemical Engineering, Xi’an University of Technology, Xi’an 710048, China; 17391831189@163.com (Z.H.); rjt15101138113@163.com (J.R.); 15760053978@163.com (J.O.); hxh@xaut.edu.cn (X.H.); wz@xaut.edu.cn (Z.W.); 2 School of Chemical Engineering, University of Chinese Academy of Sciences, Beijing 100049, China; tanbin24@ipe.ac.cn; 3 Zhengzhou Institute of Emerging Industrial Technology, Zhengzhou 450000, China; wzhang@ipezz.ac.cn; 4 School of Materials Science and Engineering, Zhengzhou University, Zhengzhou 450001, China

**Keywords:** cellulose ionogels, structure–property relationships, in situ characterization, theoretical simulation, multiscale dynamics

## Abstract

Cellulose ionogels have emerged as promising functional soft materials for flexible electronics, energy storage, and biosensing owing to their inherent biocompatibility and unique ionic conductivity. However, establishing precise structure–property relationships remains a fundamental challenge due to the complex, non-equilibrium dynamic processes—such as transient solvation, competing hydrogen-bonding networks, and mesoscopic phase separation—that occur during dissolution and gelation. Traditional static and post-mortem characterizations fail to capture these spatiotemporally dynamic behaviors, creating a critical knowledge gap. To overcome this bottleneck, the integration of real-time in situ/operando characterization techniques with multiscale computational simulations has established a novel, synergistic paradigm. This review comprehensively synthesizes recent advances in decoding the multiscale architectures of cellulose ionogels. We systematically analyze how molecular-scale calculations and time-resolved vibrational/electronic spectroscopies reveal interfacial solvation mechanisms and dynamic bond cleavage/reconstruction. We further evaluate how mesoscopic scattering, nanomechanical mapping, and rheological tools resolve network topology and structural heterogeneity. By bridging these multiscale diagnostics with macroscopic transport and mechanics, the dynamic coupling/decoupling mechanisms governing ionic conductivity, mechanical toughness, and thermal stability are critically decoded. Finally, key technical bottlenecks and future trajectories—including physics-informed machine learning, operando multi-field coupling probes, and AI-driven inverse material design—are outlined, providing theoretical guidelines and technical blueprints for next-generation sustainable ionogels.

## 1. Introduction

In recent years, natural polymer-based functional materials have emerged as a highly promising platform for sustainable energy and flexible electronics [1,2,3]. Among these, cellulose ionogels—which synergistically merge the biodegradable backbone of natural cellulose with the unique, highly conductive ionic networks of ionic liquids (ILs)—have garnered intensive research interest owing to their exceptional flexibility, outstanding ionic conductivity, structural designability, and reconfigurability [4,5,6]. Typically fabricated via anti-solvent induction or physical cross-linking, these three-dimensional polymeric networks are engineered by dissolving cellulose in tailored ILs [7,8]. Precise regulation of the underlying hydrogen-bonding (H-bonding) networks and self-assembly behaviors enables the development of cellulose-based materials with highly dynamic architectures and reversible, stimulus-responsive properties [9,10]. Recently, significant breakthroughs have been achieved in constructing high-performance cellulosic ionic materials. For instance, Jiang et al. [11] demonstrated a molecular network reconstruction strategy in cellulose ionogels by strengthening hydrogen bonds (H-bonds) and weakening electrostatic adsorption, achieving a simultaneous enhancement in tensile strength (3.5 MPa), ionic conductivity (14.3 mS cm^−1^), and thermal stability up to 120 °C. Similarly, Zhao et al. [12] developed a solvent-induced peeling strategy to fabricate ultrathin (12.46 µm) freestanding cellulose ionogel devices with high pressure sensitivity and low interfacial impedance. Furthermore, hierarchical cellulose architectures have been increasingly leveraged to program macroscale geometry and electronic functionalities in soft electronics and origami devices [13].

Despite these vast potentials, a fundamental bottleneck in the rational design and performance optimization of high-performance cellulose ionogels lies in capturing and understanding the highly dynamic structural transitions that occur during dissolution, regeneration, gelation, and multi-field operation. Traditional characterization methods are largely limited to static, post-mortem analyses, struggling to resolve these real-time, non-equilibrium processes. For instance, experimentally, Wei et al. [14] from our team demonstrated that the cellulose dissolution and regeneration pathway is governed by the highly dynamic evolution of intricate, competing H-bonding networks. Resolving these sequential changes remains exceptionally challenging using conventional infrared (IR) spectroscopy due to severe spectral peak overlaps. Furthermore, as highlighted by Lins et al. [15], the dissolving system behaves as a heterogeneous solid–liquid mixture, which impairs conventional liquid-state nuclear magnetic resonance (NMR) spectroscopy through pronounced line broadening. Parallel to these experimental hurdles, establishing accurate theoretical models for cellulose–IL systems poses distinct challenges: quantum chemical calculations offer superior atomistic accuracy but suffer from prohibitive computational costs when scaled to multi-component systems, whereas classical molecular dynamics (MD) simulations frequently lack high-precision force field parameters specifically tailored for complex cellulose–IL–solvent interactions [16]. Therefore, there is an urgent need for a unified, predictive framework that bridges multiscale in situ characterization with advanced theoretical modeling.

To emphasize the necessity and unique contribution of this review, it is essential to distinguish our work from existing review literature in related domains:

Comparison with IL-cellulose processing reviews: Existing processing-focused reviews [17,18] primarily catalog chemical dissolution, solvent basicity, and coagulation bath thermodynamics, but rarely touch upon real-time operando structural tracking or macroscopic transport-mechanics decoupling.

Comparison with application-driven soft material reviews: Literature centered on flexible electronics or hydrogel/ionogel applications [19,20] heavily emphasizes device configurations and macroscopic sensor/battery outputs, treating the internal gel network as a static “black box.”

Comparison with standalone computational reviews: Computational reviews on cellulose [21] and standalone simulation studies [22] detail atomistic Density Functional Theory (DFT) or MD algorithms in isolation, lacking direct cross-validation with real-time experimental diagnostics of dynamic bonding [23].

In contrast, this review establishes a synergistic multiscale paradigm. We systematically link molecular-scale H-bond dynamics and interfacial solvation shell configurations directly to mesoscopic network topology and macroscopic ion-transport/mechanical synergy, a conceptual framework essential for designing dynamic, sustainable polymeric materials [24]. By establishing this closed-loop “structure–method–property” blueprint, this review advances the field from empirical trial-and-error toward rational, predictive material design.

Literature Search Methodology: To ensure comprehensive coverage, transparency, and reproducibility, a systematic literature search was conducted across Web of Science, Scopus, and Google Scholar for publications from 2015 to 2026. Keywords and Boolean operators included: (“cellulose ionogel” OR “cellulosic ionic gel”) AND (“in situ characterization” OR “operando” OR “2D-COS FTIR” OR “synchrotron SAXS/WAXS”) AND (“molecular dynamics” OR “density functional theory” OR “multiscale modeling”). Literature was selected based on the following inclusion criteria: (i) studies providing mechanistic insights into non-equilibrium solvation or gelation; (ii) work deploying advanced in situ/operando experimental diagnostics; and (iii) reports linking microscopic interactions to macroscopic transport or mechanical properties. Studies focusing solely on routine material synthesis without structural or computational diagnostics were excluded.

Motivated by these advancements, this review provides a logical, bottom-up narrative tracing the multiscale lifecycle of cellulose ionogels. We begin by analyzing the intrinsic structural complexity and non-equilibrium phase transitions of the cellulose matrix (Section 2). We then detail molecular-scale solvation and H-bond dynamics through theoretical simulations (Section 3) and in situ spectroscopic techniques (Section 4). Scaling up, Section 5 explores mesoscopic network topology, microphase separation, and nanomechanics using synchrotron scattering, microscopy, and rheology. Section 6 elucidates how these micro-to-meso structures directly dictate macroscopic ionic transport kinetics and dynamic mechanical responses. Finally, Section 7 critically discusses existing bottlenecks and highlights prospective AI-assisted inverse design strategies.

## 2. Intrinsic Structural Complexity and Dynamic Phase Transitions

As the most abundant natural polymer on Earth, cellulose consists of linear macromolecular chains of D-glucopyranose units linked via *β* (1→4) glycosidic bonds [25,26]. Each repeating anhydroglucose unit possesses three reactive hydroxyl groups that participate in intricate intramolecular and intermolecular H-bonding networks [27]. These networks endow the bulk polymer with exceptional mechanical strength and provide versatile active sites for chemical functionalization [28,29]. As an emerging class of sustainable functional soft matter, cellulose ionogels synergistically combine the renewability and biodegradability of natural cellulose with the versatile ionic conductivity and non-volatility of ILs, exhibiting multiscale tunable architectures and versatile functionality [30].

### 2.1. Hierarchical Architecture and Fibril Deconstruction

Cellulose is inherently a heterogeneous polymer characterized by dense H-bonding networks between its molecular chains, where rigid chains coexist within crystalline and amorphous domains to form a multiscale hierarchical structure. Ionogels are typically fabricated using ILs, which selectively disrupt this H-bond network to facilitate dissolution while simultaneously regulating phase separation and the resulting matrix microstructure [31]. ILs exert strong plasticizing effects that induce the local unwinding of cellulose fibrils, occasionally leading to the formation of liquid-crystalline intermediate states [32]. By modifying the anion species and the overall solvent composition, the pore sizes and polymer aggregation patterns can be precisely regulated [33]. Consequently, gelation represents a typical non-equilibrium transition featuring localized H-bond reorganization and the establishment of long-range polymeric networks. Ultimately, cellulose ionogels possess an anisotropic microstructure wherein the mechanical properties, electrical conductivity, and stimulus-responsive characteristics are intrinsically coupled, enabling precise, application-tailored functional design [34].

The multi-level microstructure of the cellulose matrix serves as the fundamental basis for elucidating the structure–property relationships of these soft materials. In natural cellulose, individual molecular chains associate via intermolecular H-bonds, self-assembling into nanoscale microfibrils that comprise both highly ordered crystalline regions and structurally disordered amorphous domains [35]. Further aggregation and aligned stacking of these microfibrils lead to the formation of micrometer-scale macrofibrillar bundles and hierarchical porous networks within the fibers [36]. This multiscale aggregation is highly dynamic in IL media: the constituent anions and cations of the IL actively interact with the surface hydroxyl groups of cellulose. This interaction disrupts the native interchain H-bonds, compromising the crystalline lattices and driving the swelling of the amorphous regions [37].

**Figure 1:**
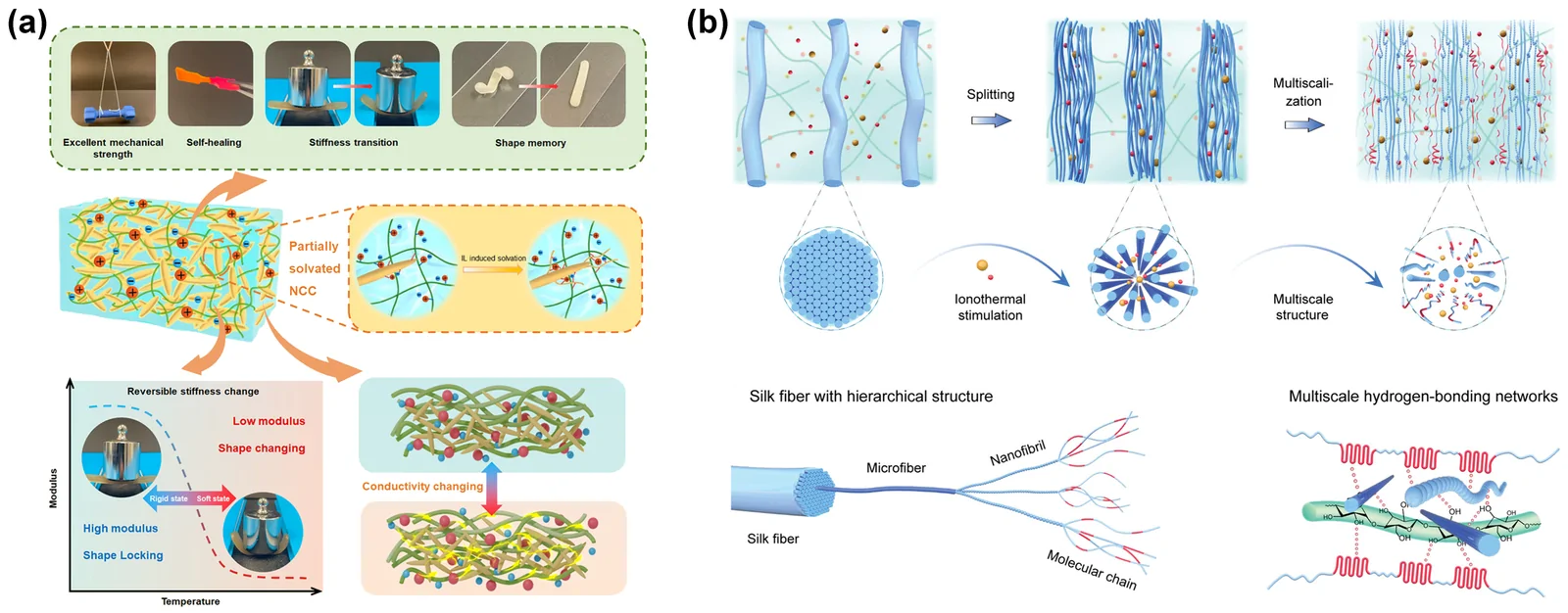
(**a**) Schematic illustration of the fabrication strategy and properties of partially solvated nanocrystalline cellulose ionogels. Reprinted with permission from [38]. Copyright 2023, Elsevier. (**b**) Creating the M-gel by inducing silk fibers into the Cel-gel matrix. Reprinted with permission from [39]. Copyright 2023, John Wiley and Sons.

Shan et al. [38] demonstrated that by controlling the swelling duration of nanocrystalline cellulose in ILs, partially solvated nanocrystalline cellulose can be obtained. In this system, the core of the crystalline domains remains intact to provide structural rigidity, while the solvated surface molecular chains confer flexibility and facilitate energy dissipation (Figure 1a). Cao et al. [39] further demonstrated that the in situ introduction of silk fibers into a cellulose–IL matrix triggers their progressive fragmentation into microfilaments, nanofilaments, and ultimately isolated molecular chains. This process establishes a multi-level H-bonded topological network spanning from molecular scales to micrometer-scale fibers (Figure 1b). These studies collectively underscore that the multi-layered architecture of cellulose exhibits significant tunability, and its responsive behavior within various IL environments dictates the macroscopic mechanical and functional performance of the resulting ionogels.

### 2.2. Solvation Mechanisms and Multifunctional Roles of ILs

To provide a fundamental, mechanistic understanding of how ILs deconstruct the cellulose matrix, cellulose–IL interactions can be deconvoluted into four distinct, interrelated microscopic mechanisms [40]:

Hydrogen Bonding Disruption Mechanism: The primary step in cellulose dissolution is the competitive disruption of native inter- and intramolecular H-bonds. Strongly basic IL anions (e.g., [OAc]^−^, [Cl]^−^, [DEP]^−^) act as potent H-bond acceptors that nucleophilically attack the hydroxyl protons of cellulose, forming strong Cellulose-OH…Anion H-bonds. This action systematically cleaves native interchain O_6_H⋯O_3_^′^ and intramolecular O_3_H⋯O_5_ H-bonds, liberating individual polymer chains from the crystalline lattice [41,42].

Ion Coordination Dynamics: Anion and cation species exhibit specific, concentration-dependent coordination modes with cellulose hydroxyls. At dilute cellulose concentrations (<25 mol%), anions coordinate with hydroxyl groups in a predominant 1:1 non-bridging mode. At elevated cellulose concentrations (>25–40 mol%), “anion bridging” emerges, where a single anion simultaneously interacts with multiple OH groups across adjacent cellulose chains [43]. Furthermore, during hydration or regeneration, the dynamic equilibrium shifts from Contact Ion Pairs (CIPs) to Solvent-Separated Ion Pairs (SIPs), significantly weakening ion-cellulose binding affinity and triggering coagulation [44].

Solvation Sheath Formation (Dual-Solvation): Complete dissolution requires a synergistic dual-solvation mechanism. While basic anions solvate the hydrophilic hydroxyl faces via strong H-bonding, bulky IL cations (e.g., [EMIM]^+^, [BMIM]^+^) intercalate between hydrophobic pyranose ring faces, stabilizing dissociated segments through van der Waals and C-H…π interactions [45]. This dual action forms a structured core–shell solvation sheath around individual cellulose chains, creating steric hindrance and effective surface charge repulsion that prevents chain re-agglomeration.

Chain Disentanglement and Fibril Unwinding: As IL ions penetrate the fibrillar crystal lattice and establish solvation sheaths, the cooperative sliding and bending of freed terminal segments induce chain disentanglement. Fibril bundles progressively unwind into individual extended worm-like chains. This physical disentanglement lowers solution viscosity and provides the molecular precursor state necessary for subsequent non-equilibrium sol-to-gel network assembly [46].

Atomistic MD simulations by Hua et al. [47] on glycine-based cationic ILs confirmed this competitive equilibrium, showing anion interaction strength following [CH_3_COO]^−^ > [Im]^−^ > [SCN]^−^ > Cl^−^ > [PF_6_]^−^. When anion–cellulose interactions dominate, cation coordination is weakened, confirming the decisive role of anion H-bond basicity (*β*) and molecular volume in governing dissolution kinetics.

### 2.3. Non-Equilibrium Phase Transitions and Gelation Kinetics

The formation of cellulose ionogels is governed by a typical non-equilibrium phase transition, wherein the system progressively evolves from a flowable sol to a structurally stable gel [48]. The kinetic pathway of this gelation process is strongly modulated by temperature gradients and external physical fields, which collectively dictate the final microstructure and macroscopic properties of the gel.

Temperature serves as a key thermodynamic parameter governing gelation kinetics. Kikuchi et al. [49] prepared cellulose hydrogels using a 1-butyl-3-methylimidazolium acetate ([BMIM][OAc])/DMSO solvent system across a temperature range of 5–60 °C. They observed that the gel shrinkage rate, water content, mechanical strength, and crystallinity exhibit highly coordinated, bimodal transitions with an inflection point at approximately 36 °C. Below 36 °C, strong anion–cation interactions coupled with weak cellulose–IL interactions favor the aggregation of cellulose chains, yielding hydrogels with high crystallinity and modulus (approximately 1.2 MPa); above this threshold, the opposite thermodynamic behavior dominates.

External physical fields can also redirect the evolution path of these non-equilibrium states. Utilizing an ultrasonic-coupled rheometer, Noguchi et al. [50] demonstrated that under 43 kHz ultrasound irradiation, the storage modulus (*G*’) of a cellulose hydrogel decreased reversibly from 4.2 × 10^4^ Pa to 4.0 × 10^3^ Pa within five minutes, recovering to its initial value upon cessation of the ultrasound. This acoustic excitation induces localized rearrangement within the gel by temporarily disrupting the H-bonding networks, an effect that is particularly pronounced in low-density networks. By modulating these intermolecular interactions, temperature gradients and external fields can thus be leveraged to achieve precise control over the macroscopic properties of ionogels under non-equilibrium conditions.

### 2.4. Processing-Induced Degradation and Thermal Stability of Cellulose in ILs

While optimizing the dissolution and network assembly of cellulose ionogels is critical, maintaining the macromolecular integrity of the cellulose backbone during thermal processing remains a pivotal challenge. Elevated processing temperatures and prolonged dissolution times often trigger severe polymer degradation within IL media [51]. This degradation not only reduces the molecular weight of cellulose but also compromises the long-term mechanical strength and electrochemical endurance of the resulting ionogels, making the analysis of degradation kinetics indispensable for rational material design [52].

From an experimental perspective, Wei et al. [53] from our team investigated the degradation behavior of wood pulp cellulose in phosphate-based ILs. They observed that the severity of polymer degradation escalated with elevated dissolution temperatures and prolonged processing times, with the degradation efficiency following the order of: [Emim]DEP > [Beim]DEP > [Emim]DMP. To resolve the underlying chemical mechanism, quantum chemical calculations were conducted on the corresponding IL–glucose binary model systems. The calculated molecular interaction strengths and reaction energy barriers corroborated the experimental degradation trend, confirming that the nucleophilic attack of phosphate anions on the glycosidic bonds is highly sensitive to the alkyl substituents on both cations and anions. These findings demonstrate that by integrating computational modeling of degradation pathways with in situ experimental diagnostics, the processing window of cellulose ionogels can be precisely tailored to suppress degradation, thereby preserving the macroscale network elasticity and stabilizing the electrochemical interfaces.

## 3. Molecular-Scale Simulations of Solvation and Interfacial Interactions

Rationally designing high-performance cellulose ionogels requires a fundamental, atomistic understanding of the complex, competing non-covalent interactions—including inter- and intramolecular H-bonding, electrostatic forces, and van der Waals interactions—that govern dissolution, network assembly, and ion transport. To complement experimental observations, computational and theoretical methodologies have emerged as indispensable tools to probe these multi-component systems across disparate spatiotemporal scales. A comprehensive computational architecture typically employs a hierarchical, multiscale strategy: high-throughput thermodynamic screening evaluates bulk solvent miscible behavior, quantum chemical calculations elucidate electronic structures and specific coordination modes, classical particle simulations track continuous structural trajectories and transport kinetics, and continuum models capture macroscale stress distribution. This section systematically reviews these three pivotal computational pillars—Conductor-like Screening Model for Real Solvents (COSMO-RS), DFT, and MD simulations—highlighting their synergistic roles in deciphering the molecular mechanisms underlying cellulose-IL interactions. To provide a clear comparative framework for choosing appropriate simulation tools in ionogel research, Table 1 systematically compares COSMO-RS, DFT, Classical MD, Coarse-Grained MD (CG-MD), Finite Element Analysis (FEA), and emerging Machine Learning Potentials (MLP) in terms of their spatial/temporal scales, predictive capabilities, computational costs, core assumptions, and intrinsic limitations.

### 3.1. Thermodynamic Screening via COSMO-RS

COSMO-RS integrates quantum chemistry and statistical thermodynamics to predict the solubility of ILs in cellulose using *lnγ* and *H^E^*, with lower values indicating stronger interactions; *lnγ* varies significantly with anion type and shows a clear negative correlation with cellulose solubility [58]. COSMO-RS also predicts solubility based on σ-profile and activity coefficient: a more right-skewed anion σ-profile indicates greater H-bond acceptance capacity. In contrast, the fiber triose activity coefficient is inversely proportional to solubility. However, this method considers hydroxyl groups as a whole, neglecting the H-bonding contribution of oxygen atoms themselves, which introduces certain limitations [59]. This reveals the thermodynamic nature of interactions between ILs and cellulose molecules [60].

**Figure 2:**
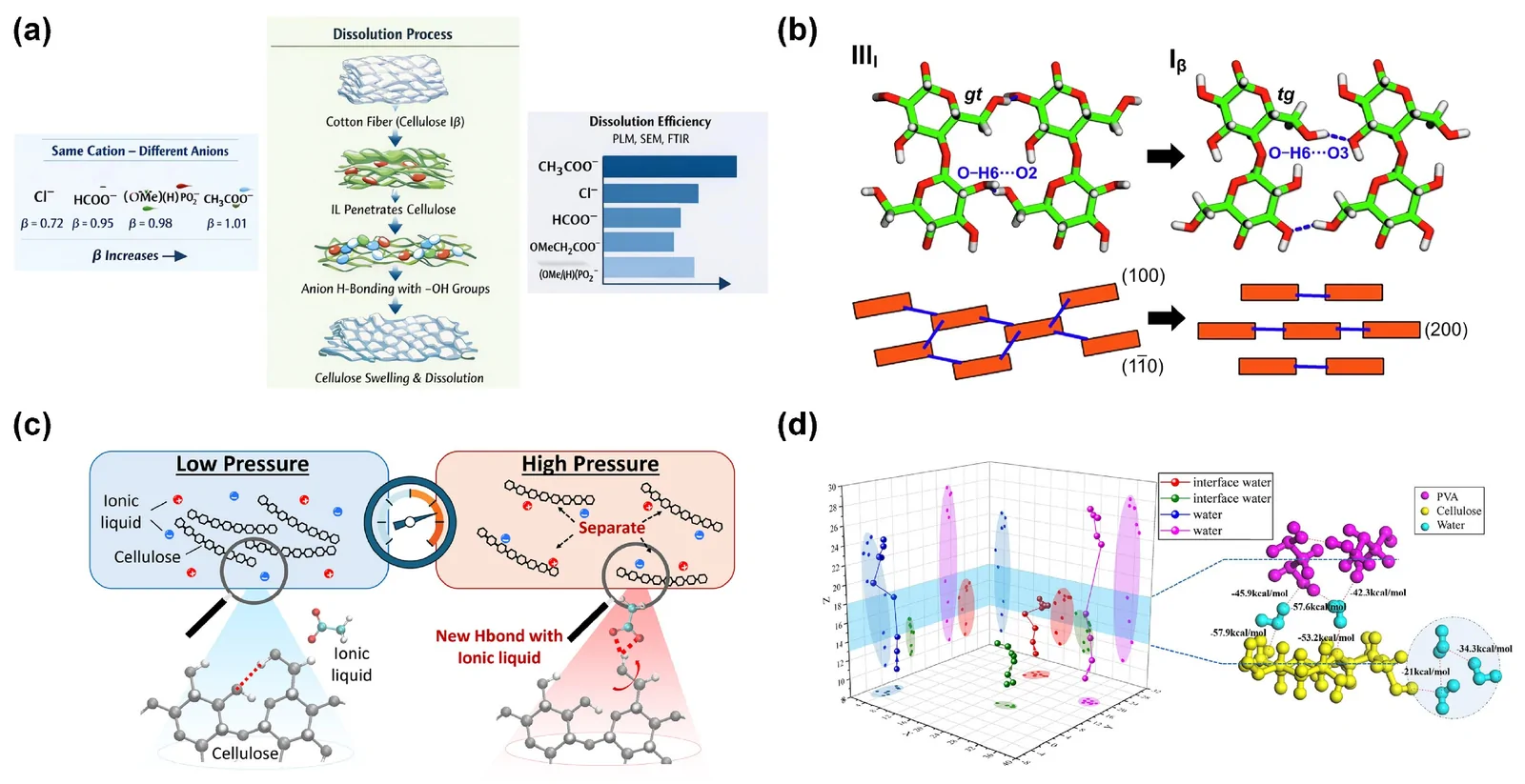
(**a**) The impact of the H-bond basicity of ILs on cotton cellulose dissolution. Reprinted with permission from [18]. Copyright 2026, MDPI. (**b**) Conversion of the molecular chain sheets with the exchange of intermolecular H-bonds during the crystal transition from cellulose IIII to cellulose I under hydrothermal conditions. Reprinted with permission from [64]. Copyright 2024, Springer Nature. (**c**) Prediction of cellulose solubility in ILs under high pressure using all-atom MD simulations. Reprinted with permission from [66]. Copyright 2026, RSC Publishing. (**d**) Trajectory of water molecules and H-bonding energy. Reprinted with permission from [67]. Copyright 2023, Elsevier.

Dissanayake et al. [18] systematically investigated the solubility of cotton cellulose in ILs consisting of the 1-benzyl-3-methylimidazolium ([BzMIM]^+^) cation paired with various anions. They discovered that while the experimentally measured Kamlet–Taft H-bond basicity (*β*) correlates with the electron-withdrawing capacity of the anion, this parameter alone cannot determine solubility efficiency. Through COSMO-RS analysis combined with surface charge density maps, they demonstrated that although the *β* value of the methoxyacetate anion ([MeOAc]^−^, 1.05) is higher than that of acetate (1.01), its larger molecular volume (327.41 Å^3^) induces significant steric hindrance, thereby restricting accessibility to the cellulose hydroxyl groups and lowering the actual solubility. In contrast, the smaller [OAc]^−^ (295.31 Å^3^) can more effectively penetrate cellulose crystalline domains to disrupt intermolecular H-bond networks (Figure 2a).

To bypass the limits of manual design, Qu et al. [61] generated a machine learning-based virtual library containing nearly 900 billion potential IL candidates and subsequently screened them using a cascaded workflow validated by COSMO-RS. The results demonstrated a strong linear correlation between the COSMO-RS-calculated cellulose *lnγ* values and experimental solubilities at 80 °C (R^2^ = 0.77), confirming the model’s reliability in screening novel cellulose solvents. Furthermore, Li et al. [62] from our team utilized COSMO-RS to calculate the thermodynamic properties of 425 ILs at infinite dilution, identifying 1-ethyl-3-methylimidazolium diethylphosphate ([Emim][DEP]) as the optimal candidate. When combined with DMSO to treat xylose residue, this system achieved a cellulose extraction rate of 99.3% under optimal conditions. Partial decomposition of *H^E^* revealed that anions govern H-bond reorganization by modulating the molecular surface charge density, thereby elucidating the coordination mechanisms between IL ions and cellulose chain segments. Meanwhile, Zhou et al. [63] from our team employed COSMO-RS to compare the fractionation efficiency of 378 anion-cation combinations, identifying 1,8-diazabicyclo [5.4.0]undec-7-ene acetate ([DBUH][OAc]) for the efficient separation of cellulose and lignin from corn stalks. Their σ-potential calculations demonstrated that the constituent anions dictate the degree of charge complementarity between the H-bond donor and acceptor regions, providing a thermodynamic framework for designing targeted IL systems compatible with cellulose ionogels.

Despite its high-throughput capability and minimal computational cost, thermodynamic screening via COSMO-RS relies strictly on the assumption of thermodynamic equilibrium in homogeneous liquid phases. Consequently, it cannot dynamically capture non-equilibrium conformational evolution, steric chain disentanglement, or transient intermediate states during gelation. Future research must integrate this thermodynamic screening approach with in situ vibrational/NMR spectroscopy and MD simulations to achieve a dynamic, multiscale analysis of H-bond reconstruction.

### 3.2. Atomistic Insights from Density Functional Theory

DFT elucidates, at the quantum chemical level, the non-covalent interactions, charge transfer behaviors, and electronic structures among ILs, antisolvents, and cellulose chains in cellulose ionogels. This method enables precise calculations of intermolecular H-bond energies and binding energies, offering unique advantages in investigating molecular-scale mechanisms, such as the coordination modes between IL ions and cellulose hydroxyl groups, as well as antisolvent-induced H-bond network reorganization.

Ju et al. [45] demonstrated via DFT calculations that the H-bonding between [OAc]^−^ and cellulose hydroxyl groups is significantly stronger than that with imidazolium cations. Conversely, when water is introduced, its high polarity competitively disrupts these cellulose-anion H-bonds, thereby promoting cellulose regeneration. Fu et al. [44] further noted that when the hydration level reaches *n* ≥ 5, the ion pairs in ILs transition from CIPs to SIPs configurations, significantly weakening their binding affinity with cellulose and triggering the coagulation cascade. Additionally, Uto et al. [64] optimized a layered molecular chain model of cellulose polymorphs using DFT, revealing distinct differences in the stability of H-bond networks between different crystal forms (cellulose I vs. II) and their consequent impacts on the dissolution kinetics (Figure 2b).

While DFT calculations provide exquisite electronic-level accuracy and precise interaction energies, they are severely restricted by system size (<200–500 atoms) and their static nature (0 K, gas phase or implicit solvent models). As a result, DFT alone cannot capture long-range cooperative chain dynamics, entropic contributions, or non-equilibrium macroscale phase separation in bulk gel networks.

### 3.3. Dynamic Trajectory Tracking via Molecular Dynamics

By solving classical equations of motion at the atomic scale, MD simulations dynamically track the diffusion trajectories of ions, polymer segments, and solvent molecules within ionogel systems, serving as a fundamental theoretical tool for elucidating ion transport kinetics. Moreover, they enable the quantitative calculation of interaction energies between the IL species and cellulose, thereby revealing the atomistic mechanisms underlying dissolution and regeneration.

Barrios et al. [65] used MD simulations coupled with RDF analysis to reveal that elevated temperatures disrupt the H-bond network in the cellulose hydration layer, reducing the local solvation energy by 22%, thereby providing key insights for optimizing ionogel processing temperatures. Meanwhile, Kikuchi et al. [66] demonstrated via MD simulations that under high pressure, the hydroxymethyl group at the C_6_ position of cellulose undergoes a conformational transition, switching the intramolecular O_6_–H⋯O_3_ H-bond to an intermolecular bond with the [OAc]^−^, which subsequently enhances the solvation capacity of the IL (Figure 2c). Sun et al. [67] employed MD simulations to track water molecule migration at the cellulose/polyvinyl alcohol (PVA) interface, revealing that water molecules form stable cellulose–water–PVA H-bridge structures that strengthen interfacial adhesion, offering valuable guidelines for solvent-mediated interfacial reinforcement in composite ionogels (Figure 2d).

Classical MD simulations bridge the gap between static quantum calculations and mesoscopic observations, accommodating spatial scales up to tens of nanometers and timescales up to hundreds of nanoseconds [68]. However, classical MD suffers from two main limitations: (i) reliance on empirical force field parameterization, which often lacks explicit electronic polarization and fixed charge transfer effects; and (ii) inability to model dynamic covalent bond breaking/formation.

To overcome these spatiotemporal and accuracy trade-offs, two emerging modeling paradigms are playing an increasingly crucial role:

Coarse-Grained MD (CG-MD): By grouping groups of atoms into single “beads” (e.g., Martini force fields), CG-MD extends simulation timescales to microseconds and length scales to hundreds of nanometers. This enables the direct observation of non-equilibrium sol-to-gel transitions, microphase separation, and polymer chain entanglement during ionogelation [69].

Machine Learning Potentials (MLP): By training neural networks or Gaussian process regression models on high-level DFT datasets, MLPs achieve quantum-chemical accuracy at computational costs comparable to classical MD. This hybrid approach enables the dynamic simulation of dynamic H-bond breaking/forming and charge transfer kinetics over extended timescales, representing a transformative direction for high-fidelity ionogel modeling [70].

## 4. Molecular-Scale In Situ Characterization of Dynamic Bond Networks

While multiscale theoretical simulations offer crucial a priori insights into molecular interaction mechanisms, directly capturing the highly dynamic, non-equilibrium structural transitions and transient solvation states of cellulose ionogels during processing or operation requires real-time, non-destructive experimental diagnostics. Molecular-scale in situ and operando characterization techniques bridge this gap by enabling the direct observation of molecular-level perturbations, chemical bond cleavage/reconstruction, and interfacial electronic shifts without introducing artifacts from sample preparation or post-mortem destruction. To provide a clear method-selection roadmap, Table 2 categorizes major characterization techniques according to their temporal/spatial resolution, measurable parameters, and principal limitations.

### 4.1. In Situ Fourier-Transform Infrared (FTIR) and Two-Dimensional Correlation Spectroscopy (2D-COS)

In situ FTIR spectroscopy enables the direct, real-time observation of vibrational perturbations within polar functional groups (such as hydroxyl and carbonyl groups) of cellulose ionogels, facilitating the detailed analysis of internal H-bonding dynamics, ion-coordination environments, and intermolecular interactions. To resolve the complex, overlapping spectral bands of these networks, 2D-COS is employed to distinguish the sequential response behaviors and cleavage sequences of different functional groups under external physical perturbations, thereby exposing the molecular-scale dynamic evolution of the gel network [71].

**Figure 3:**
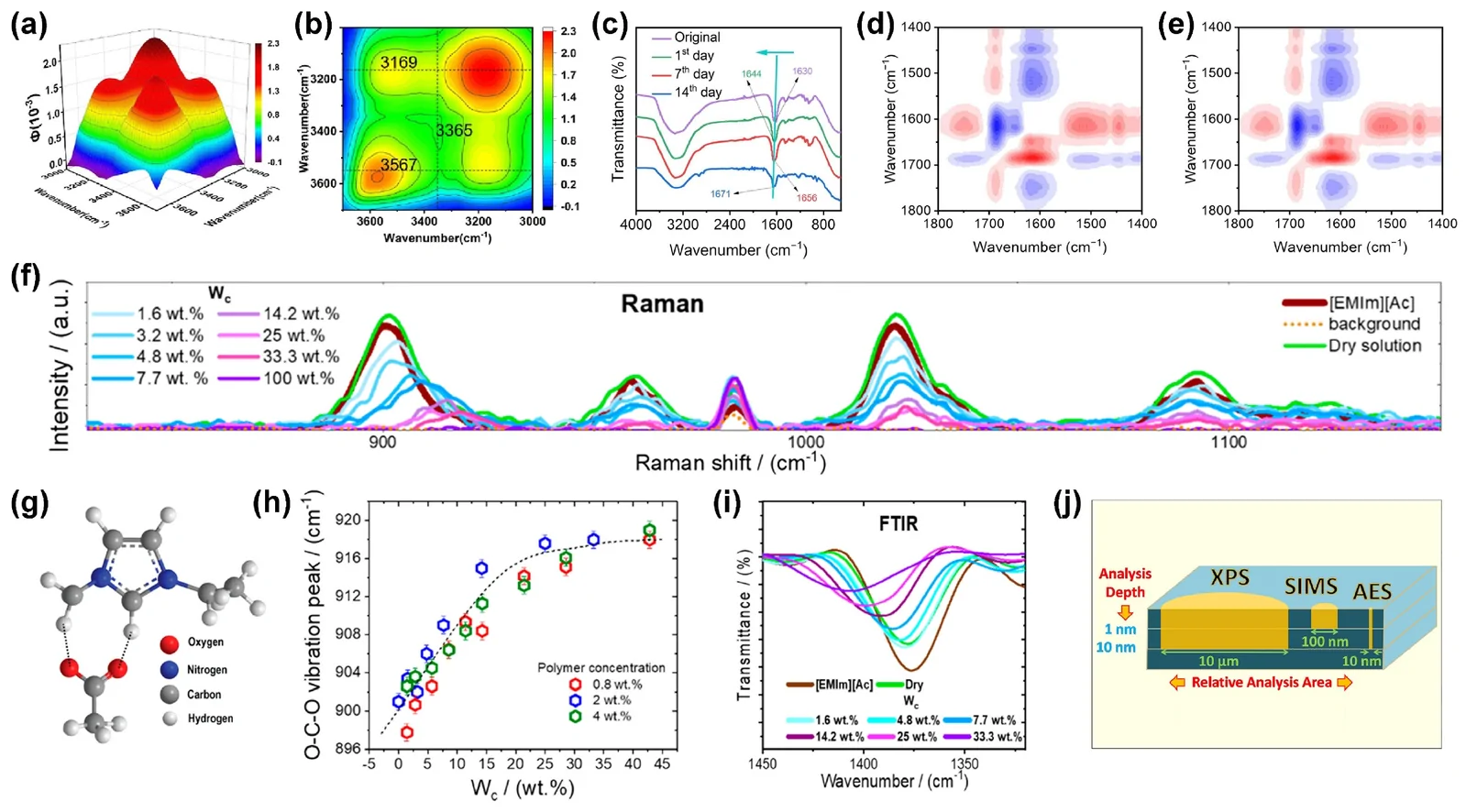
The two-dimensional composition-dependent FTIR spectra of DES-1-treated CNFs in the range of 3700–3000 cm^−1^. (**a**) Synchronous 3-D correlation spectra. (**b**) Synchronous 2-D correlation spectra. Reprinted with permission from [81]. Copyright 2023, Elsevier. Characterization of the H-bonds and the structural changes during the swelling process. (**c**) FTIR spectra of Ion-C-P(AA-co-AAm) hydrogels with diverse immersion times (the arrow indicates the characteristic peak shift). 2DCOS (**d**) synchronous and (**e**) asynchronous contour plots obtained from the shige analysis of the FTIR spectra (red and blue colors represent positive and negative correlation intensities, respectively). Reprinted with permission from [82]. Copyright 2024, Elsevier. (**f**) Raman spectra for a 2 wt % native cellulose/EMImAc solution at various water contents. (**g**) EMImAc chemical structure. (**h**) O−C−O molecular vibration as a function of water concentration *W_c_* for three different polymer concentrations. (**i**) FTIR for a 2 wt % native cellulose/EMImAc solution at various water contents. Reprinted with permission from [72]. Copyright 2024, ACS Publications. (**j**) Schematic representation of the three primary surface characterization techniques and their relative area of analysis and analysis depth (arrows indicate the analysis depth and relative analysis area). Reprinted with permission from [87]. Copyright 2022, Elsevier.

As demonstrated by Chen et al. [81] using 2D-COS FTIR on cellulose nanofibrils (CNFs) treated with deep eutectic solvents, the asynchronous correlation maps reveal a strict, time-resolved evolutionary cascade of H-bond breaking. Specifically, the intramolecular O_3_H⋯O_5_ bonds undergo initial cleavage due to solvent penetration, followed sequentially by the cleavage of intermolecular O_6_H⋯O_3_^′^ bonds, and finally the reorganization of intramolecular O_2_H⋯O_6_ bonds (Figure 3a,b). Similarly, Endo et al. [43] captured the competitive coordination dynamics between IL ions and cellulose hydroxyls, demonstrating that the redshift of the -OH stretching vibration (∼3300 cm^−1^) directly tracks the localized disruption of native crystalline lattices, while the subsequent blueshift during antisolvent addition signals the reconstruction of locally ordered H-bonded junctions during sol-to-gel transition. To address mechanical stability during swelling, Zhang et al. [82] mapped the stepwise formation of newly generated H-bonding junctions, providing thermodynamic proof for swelling resistance (Figure 3c–e).

Although 2D-COS FTIR offers peerless sensitivity in tracking dynamic H-bond breaking and forming, it suffers from intrinsic limitations. It cannot resolve 3D spatial network architectures or local spatial heterogeneity. Furthermore, data interpretation requires careful baseline correction and deconvolution to avoid artifactual cross-peaks. Future research should integrate 2D-COS with MD simulations and synchrotron scattering under synchronous force-electricity-spectroscopy coupling [83].

### 4.2. In Situ Raman Spectroscopy

In situ Raman spectroscopy provides complementary vibrational insights to infrared techniques, demonstrating high sensitivity to homopolar bonds, skeletal vibrations, and local ring-stacking environments of ILs within polymer matrices. In cellulose–IL systems, Raman spectroscopy is widely utilized to track the coordination state of imidazolium-based or organic-salt-based ILs during solvation and network construction. For instance, the stretching vibration of C-H bonds on the imidazolium ring (C_2_−H, C_4_−H, C_5_−H) exhibits measurable frequency shifts upon interacting with cellulose hydroxyl groups, directly reflecting localized H-bonding strength and the associated cation–anion–cellulose interaction dynamics.

Furthermore, in situ Raman spectroscopy offers distinct capabilities in investigating anionic coordination states during the gelation of cellulose–IL mixtures. For instance, Mohamed Yunus et al. [72] employed this technique to monitor the shift in the O–C–O vibrational mode of [OAc]^–^ during the water-induced sol–to–gel transition of native cellulose/[EMIM][OAc] solutions. They observed that the O–C–O vibration peak, originally located at ~900 cm^−1^, systematically shifted toward higher wavenumbers upon the addition of water. This spectral shift directly reflects the preferential solvation of [OAc]^−^ by water molecules, which subsequently weakens both anion–cation and anion–cellulose interactions (Figure 3f–i). This dynamic decoupling effectively releases the cellulose chains from the IL solvation shell, facilitating interchain self-association and elucidating the microscopic mechanism where water acts as an anti-solvent or “gelation initiator” to drive the reconstruction of the cellulose H-bonding network.

### 4.3. In Situ and Near-Ambient X-Ray Photoelectron Spectroscopy

For multi-component systems such as cellulose ionogels, the strength of electrostatic interactions between constituent anions and cations, as well as their coordination with the polymer backbone, can be evaluated by monitoring core-level binding energy shifts in X-ray Photoelectron Spectroscopy (XPS) [73]. Furthermore, combining principal component analysis with non-linear peak-fitting protocols enables the separation of pristine cellulose signals from radiation-induced degradation species [84]. For example, the strong solvation environments of [EMIM]^+^ and HSO_4_^−^ within ionogel matrices can be validated by monitoring chemical shifts in N 1s and S 2p core levels [85].

It should be emphasized that advanced core-level tracking and valence-band peak-fitting protocols were initially demonstrated on inorganic model surfaces (e.g., real-time gas adsorption on WS_2_ domains or TiO_2_ polymorph indexing) [86]. Translating these valence-band analysis protocols to cellulose ionogels is an adapted and prospective methodology [87]. It allows researchers to resolve selective solvent coordination across distinct crystalline and amorphous phases (Figure 3j).

Conventional high-vacuum XPS is restricted by strict vacuum requirements, making it impossible to capture hydrated or liquid-state gel dynamics. While Ambient-Pressure XPS (APXPS) overcomes this by operating at near-ambient humidity, it suffers from severe X-ray beam damage (radiation degradation of cellulose backbones under prolonged exposure), low signal-to-noise ratios through dense gas layers, and surface-bound sampling depth (<10 nm) which cannot probe buried bulk-phase transport [88,89].

## 5. Mesoscopic Network Topology and Structural Heterogeneity

Scaling up from molecular-scale non-covalent interactions, the ultimate mechanical resilience and ion-transport efficacy of cellulose ionogels are fundamentally dictated by their mesoscopic network topology, phase-separated domain morphology, and hierarchical structural heterogeneity [90]. Spanning length scales from a few nanometers to several micrometers, the mesoscopic architecture serves as a pivotal structural conduit that translates localized molecular H-bonding networks into macroscopic bulk elasticity, fracture toughness, and continuous ion-conduction pathways. To decode these complex spatial configurations, a complementary suite of mesoscopic characterization techniques is required: in situ optical and electron microscopy methods enable the direct visualization of fibrillar assembly, entanglement density, and phase-separated microdomains; synchrotron radiation scattering and diffraction techniques yield non-destructive, quantitative measurements of network mesh size, correlation lengths, and crystalline polymorphic transformations; bulk rheological analysis captures viscoelastic modulus evolution and dynamic sol-to-gel transition kinetics; and in situ atomic force microscopy (AFM) with nanomechanical mapping bridges topological morphology with spatial heterogeneity in surface elasticity and adhesion [91,92]. This section systematically outlines these mesoscopic analytical methodologies, highlighting their synergistic roles in elucidating how three-dimensional network topologies dictate the macroscopic functionality of cellulose ionogels.

### 5.1. In Situ Optical and Electron Microscopy

#### 5.1.1. In Situ Optical Microscopy

In situ optical microscopes enable the direct, real-time visualization of the topological evolution of fibrillar networks during gelation, capturing micrometer-scale morphological transformations and dynamic assembly pathways. For cellulose-based systems, these techniques resolve fiber dispersion states, entanglement densities, and network connectivity; however, due to the constraints of the optical diffraction limit, they cannot resolve sub-100 nm features, localized molecular solvation, or polymer chain conformations.

**Figure 4:**
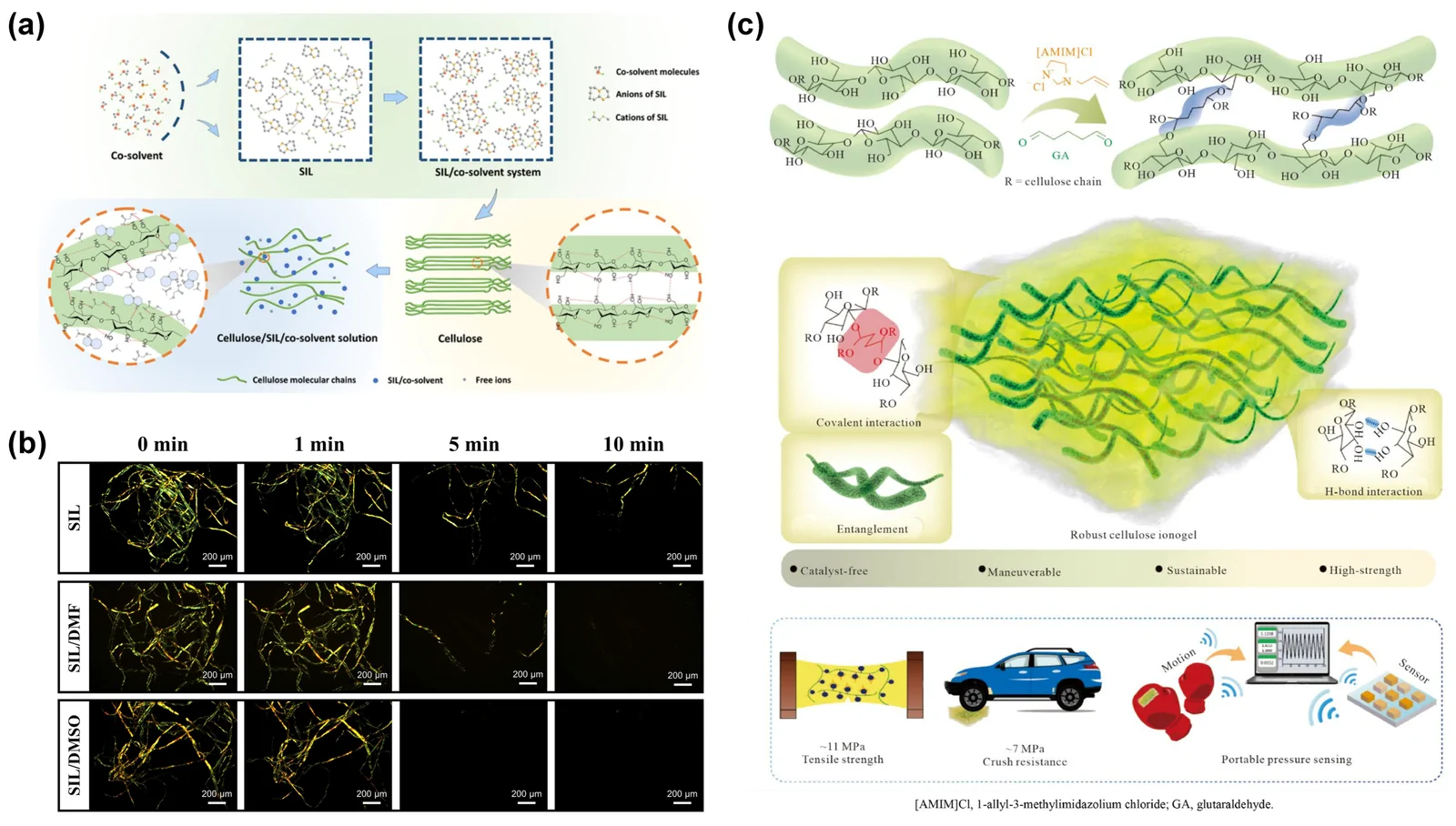
(**a**) A possible schematic diagram of co-solvents interacting with SIL during cellulose dissolution. (**b**) PLM images of DWP fibers in SIL and SIL/co-solvent systems at 0 min, 1 min, 5 min, and 10 min, respectively. Reprinted with permission from [93]. Copyright 2024, Elsevier. (**c**) Schematic illustration of the synthesis, properties, and applications of the robust cellulose ionogel. Reprinted with permission from [48]. Copyright 2025, Elsevier.

Wang et al. [93] employed polarized light microscopy (PLM) to monitor cellulose dissolution within an ultra-alkaline IL/co-solvent system, tracking the gradual disappearance of fiber birefringence at 80 °C. This study visually proved that co-solvents such as DMSO accelerate macrofibrillar swelling and subsequent chain dissociation, offering mesoscopic evidence of the structural evolution of the gel precursor (Figure 4a,b).

Despite its accessibility, in situ optical microscopy remains blind to molecular-level details, such as H-bond dynamics or localized ion-coordination environments. Consequently, it must be combined with in situ Raman spectroscopy, Small-Angle X-ray Scattering (SAXS), and MD to construct a multiscale characterization framework capable of bridging the gap between localized chemical environments and macroscopic network topology.

#### 5.1.2. Cryo-Transmission Electron Microscopy and Scanning Electron Microscopy (Cryo-TEM/SEM)

Cryo-TEM/SEM utilize rapid-freezing vitrification techniques to preserve the native, solvent-solvated structures of the cellulose network and its interfaces under near-native conditions, successfully preventing the structural collapse or artifact generation caused by conventional drying or electron-beam irradiation [94].

While Li et al. [95] demonstrated these microphase separation phenomena in acrylic-based (PHI) ionogels, combining SEM with energy-dispersive X-ray spectroscopy (EDS) elemental mapping provides a direct methodology for tracking IL distribution within multi-component networks. Their EDS mapping confirmed that the IL resides predominantly within the continuous, polymer-rich phase. At the same time, the acrylic-rich microdomains showed distinct elemental voids, proving the existence of phase-separated ion conduction channels. Translating this approach to cellulose, Yang et al. [48] integrated SEM with SAXS to show that chemical crosslinking increases electron density gradients and drives the self-assembly of robust phase-separated domains in cellulose-based ionogels, thereby enhancing their mechanical strength and fracture toughness (Figure 4c).

Nevertheless, conventional electron microscopy represents a static characterization tool constrained by high-vacuum requirements, which limits its ability to capture dynamic structural transitions in real time. Future research in this domain must look toward the integration of emerging in situ liquid-cell TEM and environmental SEM to record the real-time solvation, gelation, and interfacial ion-transport processes at the nanometer scale.

### 5.2. Synchrotron Radiation X-Ray Scattering/Diffraction

#### 5.2.1. In Situ Small-Angle X-Ray and Neutron Scattering (SAXS/SANS)

In situ SAXS and SANS, operating on the principles of electron density and scattering length density (SLD) contrast, respectively, enable the non-destructive structural characterization of fully solvated gel networks across length scales ranging from 1 to 1000 nm [96]. To probe the molecular solvation sheath and local chain conformations of dissolved cellulose prior to and during gelation, SANS with contrast variation offers unique capabilities that complement X-ray techniques. For instance, Raghuwanshi et al. [97] performed contrast-variation SANS on microcrystalline cellulose and deuterated bacterial cellulose dissolved in deuterated (IL-D14) and hydrogenated (IL-H14) [EMIM][OAc]. Surprisingly, SANS intensities from hydrogenated cellulose in deuterated IL were dramatically lower than theoretical predictions (Δ*ρ_exp_* being 1.8–2.2 times smaller than Δ*ρ_theo_*). This contrast reduction quantitatively proved the tight binding of acetate anions to cellulose hydroxyl groups (with a lower-limit molar ratio of ~0.74–0.93 acetate per anhydroglucose unit), establishing that the IL forms a solvation sheath that imparts effective charges to cellulose chains, preventing re-agglomeration in solution.

To quantitatively resolve the nanoscale spatial dimensions of this solvation sheath, Koide et al. [98] performed synchrotron SAXS on cellulose/[EMIM][OAc] and cellulose/[EMIM][DEP] solutions using a coaxial double-layer cylinder model. By analyzing radial electron distance distribution functions (*P*_*c*_(*r*)), they identified a core–shell structure where the inner cylinder core corresponds to the cellulosic chain (*r_in_* = 0.36–0.54 nm) and the outer shell represents a lower-electron-density solvent sheath (*r_out_* − *r_in_* = 0.41–0.66 nm, matching the molecular size of [EMIM][DEP] and [EMIM][OAc]). Heating was shown to break intramolecular H-bonds and induce chain conformation rearrangement into a new minimal energy state with a denser, tighter solvation layer.

Furthermore, extending SAXS measurements across the entire concentration spectrum (0–100 mol%), Endo et al. [99] investigated the nanostructural evolution of cellulose/[EMIM][OAc] mixtures. Kratky plots and generalized Guinier-Porod fitting demonstrated that at concentration regimes >25 mol%, “anion bridging” occurs (where a single acetate anion coordinates with multiple OH groups on adjacent chains). This anion bridging completely deconstructs native microfibrils at 40 mol% cellulose and induces the emergence of two distinct nanoscale scatterers: invariant 4.1 nm microfibril-like domains and 12–20 nm flexible chain entanglement aggregates. These entangled nanostructures impart mechanical moldability to lyotropic liquid-crystalline cellulose/IL composites at 30–50 mol% concentrations, providing direct mesoscopic evidence of how concentration-dependent anion coordination dictates gelation and mechanical integrity.

**Figure 5:**
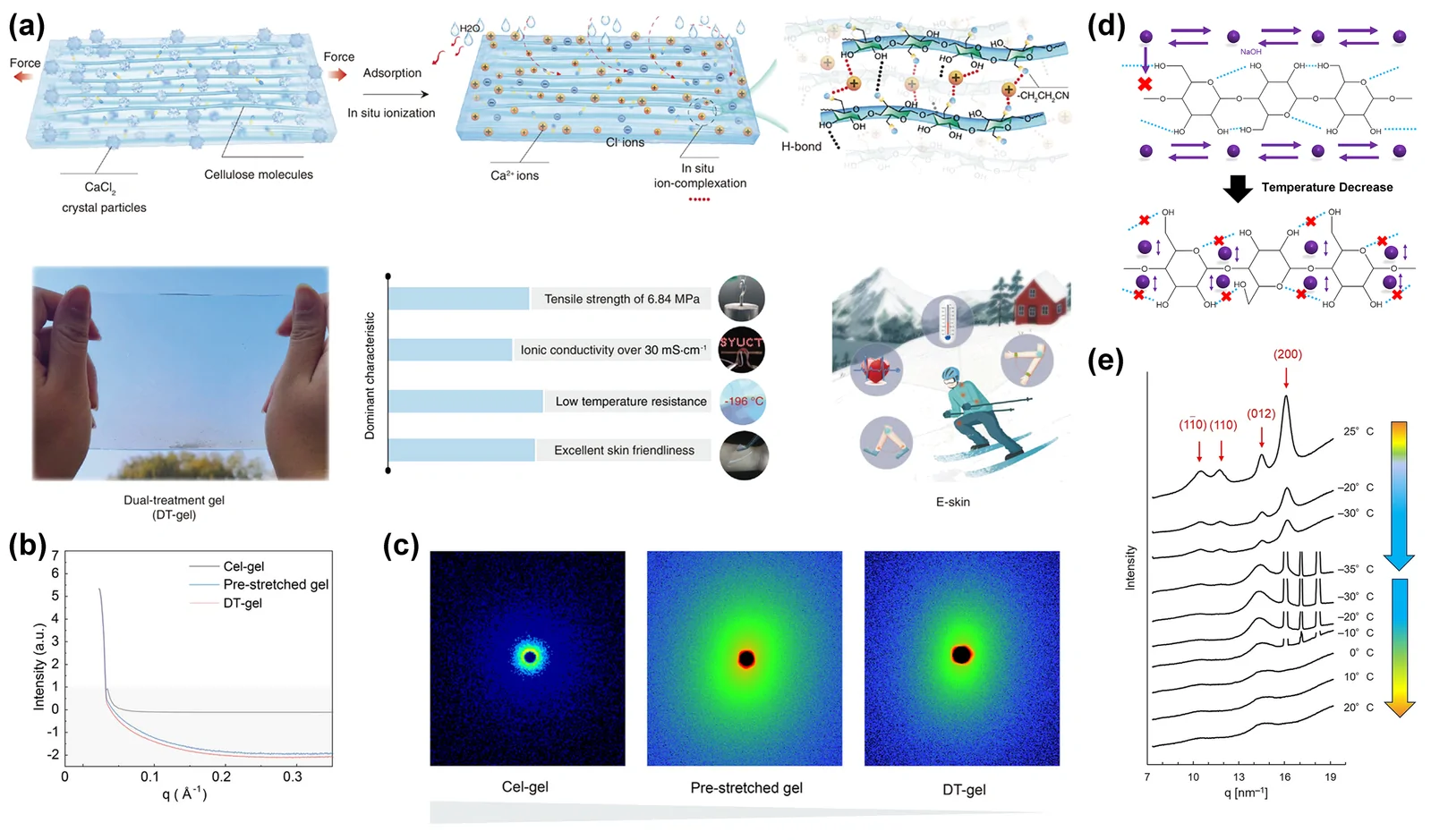
(**a**) Construction of DT-gel through synergistic mechano-ionic induced design. (**b**) SAXS curves of Cel-gel, Pre-stretched gel, and DT-gel. (**c**) Comparison of 2D SAXS diffraction patterns of Cel-gel, Pre-stretched gel, and DT-gel. Reprinted with permission from [100]. Copyright 2025, John Wiley and Sons. (**d**) Proposed mechanism for the defibrillation of the cellulose I molecular chains induced by alkali treatment at low temperatures. (**e**) Temperature-variable WAXS profiles of 10 wt% NaOH-soaked cellulose powder. Reprinted with permission from [106]. Copyright 2024, Elsevier.

In composite ionogels, Bai et al. [100] employed in situ 2D SAXS to track structural evolution from sol to solid states, demonstrating that pristine gels exhibit isotropic scattering rings, whereas tensioned samples display highly anisotropic spot patterns, confirming force-directed scaffold alignment (Figure 5a–c). Cao et al. [39] used SAXS to monitor the dynamic, heat-induced dissociation of fibroin fibers within a cellulose–IL matrix into nanofibrils. In another study, Wang et al. [101] used SAXS to show that crystallized Crystal-Cel gels exhibit a sharp scattering peak at *q_max_* = 0.024 Å^−1^ (*d* = 26.2 nm), quantitatively confirming the transition to highly ordered ion-liquid crystalline networks.

Although single-source in situ SAXS can resolve spatial parameters, it cannot distinguish specific chemical bond types from local chain dynamics. This limitation necessitates its integration with vibrational spectroscopy, rheology, and MD simulations. When combined with 2D-COS, SAXS can track the reaction sequences between cellulose hydroxyls and crosslinking agents, revealing the correlation between chemical crosslinking and physical network densification [102]. Furthermore, employing machine learning-enhanced inversion algorithms—such as the CREASE algorithm—allows the direct reconstruction of material-specific spatial network topologies and local solvent distribution profiles from raw SAXS scattering profiles, paving the way for the rational, digital design of cellulose ionogels [103].

#### 5.2.2. In Situ Wide-Angle X-Ray Scattering (WAXS)/In Situ X-Ray Diffraction (XRD)

In situ WAXS and XRD enable the real-time tracking of atomic-scale chain packing, polymorphic transformations (such as the transition from cellulose I to cellulose II), and crystallinity changes during ionogelation [104,105]. These techniques provide direct experimental evidence of the structural transitions of the crystalline lattice under environmental or mechanical stress.

Through the integration of in situ WAXS and solid-state ^13^C cross-polarization magic-angle spinning NMR, the relationship between solvent coordination and polymer chain segment rearrangement can be clearly resolved. Kugo et al. [106] used this approach to show that under low-temperature alkaline conditions, sodium ions penetrate the cellulose crystalline lattice, disrupt the intramolecular H-bonds, decrease overall crystallinity, and induce an antiparallel alignment of the polymer chains that drives the formation of the cellulose II polymorph (Figure 5d,e). Additionally, Gentile et al. [107] employed 2D in situ WAXS to isolate crystalline scattering from amorphous halos, proving that cellulose crystallinity increases with stretching ratio before reaching a stable plateau.

However, traditional WAXS/XRD techniques struggle to isolate specific H-bond dynamics within static, highly crystalline structures. This limitation requires their integration with solid-state NMR (SSNMR), rheology, and MD simulations. In the future, the integration of multiscale in situ WAXS/XRD with machine learning-assisted phase inversion models will significantly advance the automated structural indexing and rational design of cellulose-based ionogels [108].

### 5.3. Bulk Rheological Analysis

In situ rheology enables the highly sensitive monitoring of bulk viscoelastic moduli under mild, non-destructive oscillatory shear, allowing for the precise determination of gel points (*T_gel_*) and the quantification of physical crosslink densities. Furthermore, by evaluating modulus evolution across wide temperature and frequency sweeps, it facilitates the thermodynamic analysis of interfacial interactions and the reversibility of physical crosslinks within the bulk gel matrix.

**Figure 6:**
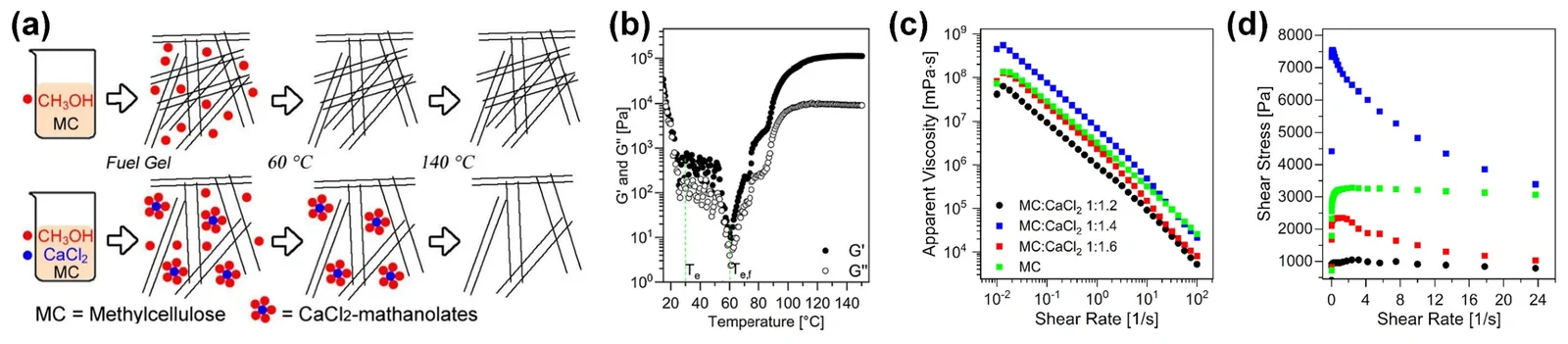
(**a**) Methylcellulose as a non-bonding gelling agent for calcium chloride methanol fuel gels. (**b**) Oscillatory temperature sweep experiments of the 22 wt% MC in methanol display G′ and G″ as a function of temperature within the linear viscoelastic regime. The flow curves of MC in methanol and MC:CaCl_2_ at three different ratios of 1:1.2, 1:1.4, and 1:1.6 in methanol are presented. The apparent viscosity (**c**) or shear stress (**d**) is plotted as a function of the shear rate at 25 °C. Reprinted with permission from [110]. Copyright 2024, Elsevier.

To establish macroscopic rheological scaling laws for cellulose–IL solutions and gels, Gericke et al. [109] systematically investigated the viscoelastic transition from dilute to concentrated regimes, demonstrating that the zero-shear viscosity (*η*_0_) exhibits a steep power-law dependency on cellulose concentration (*η*_0_*_–_*∝*c*^4.2^ in concentrated states), reflecting the transition from unentangled regimes to dense physical entanglement networks. Building on this, De Cataldo et al. [110] investigated the phase separation and gelation kinetics of methylcellulose/methanol gels, demonstrating that *G*^′^ consistently exceeds the loss modulus (*G*^″^) at elevated temperatures, confirming that the gel network is maintained by stable, entropic fiber entanglements (Figure 6). Additionally, Sen et al. [111] monitored the aging kinetics of physical hydrogels, proving that temporary, dynamic non-covalent crosslinks gradually transition into permanent covalent networks over time, manifesting as a continuous increase in *G’* and distinct shifts in stress-relaxation profiles.

While in situ rheology is exceptionally sensitive to macroscopic network transitions, it acts as a bulk averaging technique that cannot resolve the chemical nature or local coordination states of crosslinking junctions. To overcome this limitation, future studies must develop hyphenated rheological techniques—such as in situ rheo-FTIR or rheo-SAXS—to build multi-dimensional correlation models that simultaneously map macroscopic mechanical responses to microscale chemical and topological transformations.

### 5.4. In Situ Atomic Force Microscopy and Nanomechanical Mapping

AFM, particularly when operating in Peak Force Quantitative Nanomechanical Mapping (PF-QNM) mode, bridges the critical gap between mesoscopic network morphology and localized surface mechanical properties. By acquiring high-speed force-displacement curves at approximately 2 kHz, PF-QNM enables the simultaneous, high-resolution mapping of surface topography and nanomechanical properties (such as local Young’s modulus, deformation, and tip-sample adhesion) at the nanometer scale [78]. This technique offers distinct advantages over bulk mechanical testing by resolving spatial heterogeneity and phase-separation boundaries within the gel network.

**Figure 7:**
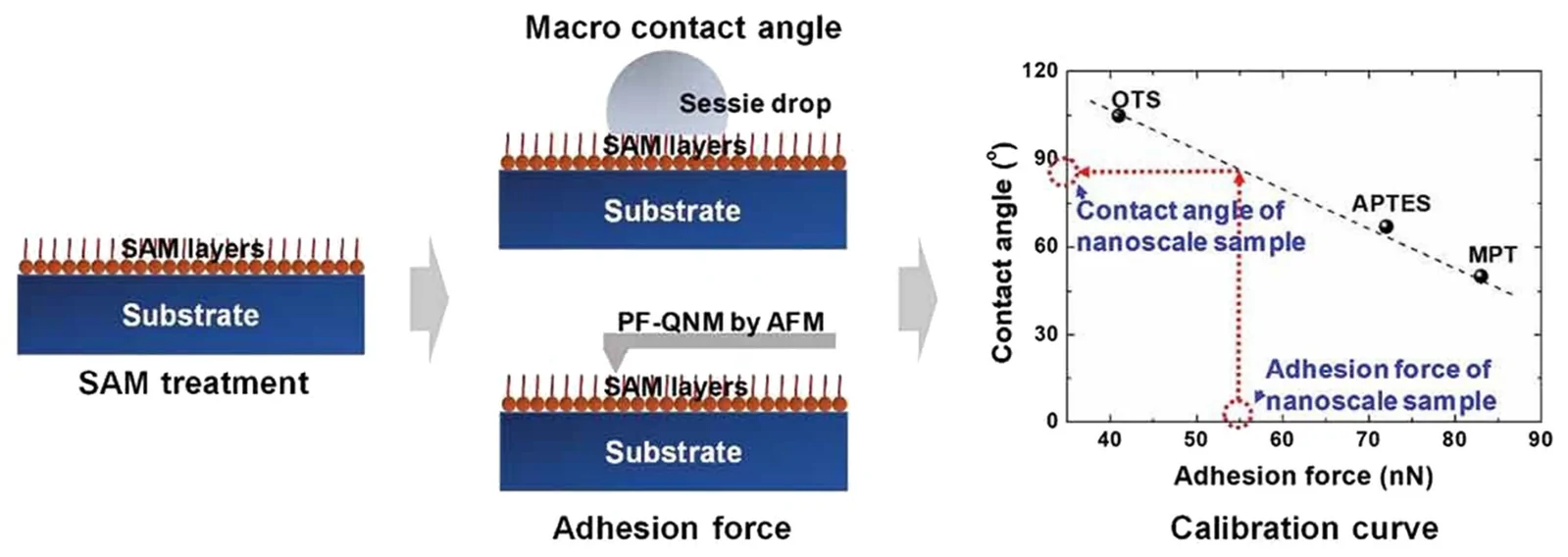
Schematic illustration of nCA measurement process using PF-QNM mode (the red dotted line and circles illustrate the derivation of the contact angle from the measured adhesion force via the calibration curve). Reprinted with permission from [112]. Copyright 2022, Taylor & Francis.

Research by Ha et al. [112] proved that measuring the tip-sample adhesion force using PF-QNM, coupled with surface energy analysis, can resolve the chemical states and interfacial interaction strengths of heterogeneous gel surfaces (Figure 7). Cellulose ionogels typically exhibit a biphasic “rigid framework–flexible channels” architecture: polymer-rich domains assemble into rigid networks (Young’s modulus 0.3 MPa) via H-bonding. In contrast, solvent-rich domains provide flexible conduits that enable high fracture strains (up to 470%) [113]. Bakonyi et al. [114] employed PF-QNM to observe the in situ formation of [BMIM]Cl/cellulose ionogel films under liquid environments, revealing that substrate confinement significantly flattens the bottom interface during gelation. Specifically, the air-exposed side of the film exhibited a roughness of R_a_ = 428 nm, whereas the glass-confined side displayed a much smoother surface with R_a_ = 222 nm. Although pores ranging from 200 nm to 2 μm were observed on the film surface, the corresponding adhesion force maps remained highly homogeneous, indicating the formation of a uniform, well-distributed mesoscopic network between the cellulose matrix and the IL.

Because PF-QNM alone cannot directly provide chemical information or track the dynamic reconstruction of buried H-bonds, it has limitations in investigating deep, buried interfacial structures. To address this, combining PF-QNM with tip-enhanced Raman spectroscopy or AFM-IR is crucial to establish nanoscale spatial-chemical-mechanical maps that elucidate how molecular-scale interfacial H-bonding networks govern macroscopic mechanical and transport behaviors.

## 6. Macroscopic Property Decoupling: Ion Transport and Mechanical Performance

A central hallmark in the design of high-performance cellulose ionogels is navigating the delicate interplay between ionic transport kinetics and mechanical strength—two macroscale properties that traditionally suffer from a classical trade-off in solid-state polymer electrolytes. In conventional solid polymer systems, increasing polymer density or crystallinity enhances mechanical strength (*E*, *σ_t_*) but severely restricts chain mobility and free volume, suppressing ionic conductivity (*σ_i_*). Conversely, increasing solvent/IL loading boosts ion mobility but results in soft, weak, or brittle gels. Driven by dynamic H-bonding networks, microphase-separated architectures, and nanostructural confinement, cellulose ionogels achieve a decoupled enhancement where high ionic conductivity coexists with exceptional mechanical toughness, elasticity, and fatigue resistance. Deciphering these dynamic coupling and decoupling mechanisms demands a multi-methodological approach bridging electrochemical kinetics and mechanics.

### 6.1. Microscopic Ion Transport Mechanisms

To establish a rigorous theoretical foundation for transport kinetics, ion conduction in cellulose ionogels can be deconvoluted into four interrelated microscopic mechanisms:

Vehicular Transport Mechanism: Mobile ions (e.g., [EMIM]^+^, [OAc]^−^, or Li^+^) migrate together with their surrounding solvation shells (composed of solvent or IL molecules) as hydrodynamic entities. Governed by the Stokes-Einstein relation (*D* = *k_B_T*/(6*πηr_H_*)), vehicular transport dominates in low-viscosity microdomains or solvent-rich continuous channels where ion displacement is constrained primarily by local hydrodynamic drag [115].

Hopping (Grothuss-Like) Mechanism: Ions jump sequentially between dynamic coordination sites (such as hydroxyl groups along cellulose chains or adjacent anion clusters) without dragging their entire solvation sheaths. This hopping process depends on the dynamic breaking and reforming of local coordination bonds. Because hopping bypasses bulk matrix viscosity constraints, it enables rapid charge migration even within mechanically rigid, highly crystalline polymer networks [116].

Polymer-Chain Segmental Mobility: In semi-crystalline or amorphous matrix regions, the local thermal fluctuation and segmental motion of cellulose chains create transient free-volume voids that facilitate ion displacement. This mechanism is closely coupled with the glass transition temperature (*T_g_*) and follows Vogel–Fulcher–Tammann (VFT) relaxation behavior, where segment mobility assists ion hopping across localized potential energy barriers [117].

Ion-Pairing and Dissociation Dynamics: The concentration of active charge carriers is governed by the equilibrium between neutral CIPs, SIPs, and fully dissociated free ions. High-dielectric additives (such as water or DMSO) or competitive coordination by cellulose OH groups promote CIP-to-SIP transitions. According to the Nernst-Einstein equation (*σ_i_* = ∑*n_j_q_j_μ_j_*), maximizing the fraction of dissociated free ions (*n_j_*) dramatically boosts ionic conductivity without altering overall gel mechanics [118].

#### 6.1.1. Broadband Dielectric Spectroscopy (BDS)

BDS is a powerful tool for capturing various polarization behaviors induced by the displacement of mobile charges under alternating electric fields, with distinct relaxation kinetics reflecting different micro-environmental interactions [80]. For instance, cellulose ionogels fabricated via in situ dissolution and self-assembly contain both mobile free ions and a highly dynamic, reversible H-bonding network. Within these systems, macroscopic ion migration and microscopic H-bond rearrangement manifest as two distinct types of polarization relaxation. Consequently, BDS enables the separate, quantitative analysis of these concurrent processes—ion transport and matrix dynamics—thereby establishing a quantitative bridge between the gel’s microstructural characteristics and its macroscopic features, such as high ionic conductivity, rapid self-healing capability, and low-temperature charge transport.

**Figure 8:**
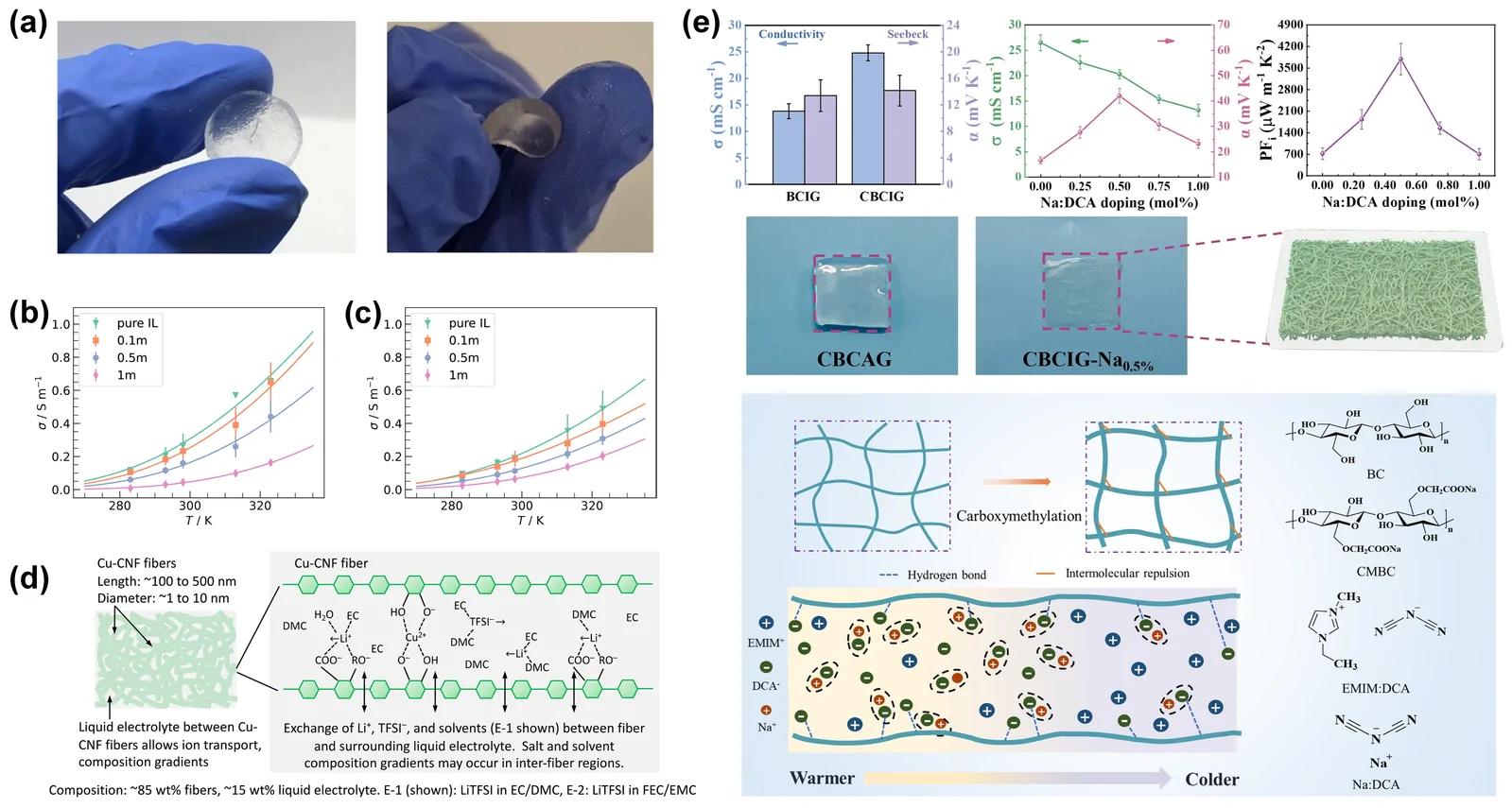
(**a**) Images of a pure cured ionogel. [C_4_C_1_Pyrr][TFSI] samples ionic conductivities in (**b**) liquid state and (**c**) PVdF-based gel. Lines correspond to VFT fits. Reprinted with permission from [120]. Copyright 2026, Elsevier. (**d**) Schematic of Cu-CNF fiber-based electrolyte structure and composition. Reprinted with permission from [123]. Copyright 2026, ECS. (**e**) Thermoelectric properties of the bacterial cellulose-based ionogels. Reprinted with permission from [126]. Copyright 2024, Elsevier.

A key strength of BDS lies in its ability to resolve how water molecules synergistically modulate ion transport and network elasticity. Wu et al. [119] demonstrated that moisture content plays a decisive role in regulating the competing H-bonds among cellulose–cellulose, cellulose–IL, and cellulose–water interfaces in all-cellulose ionogels. At an optimized water content of approximately 30 wt%, the concentration and migration rate of free ions reach a thermodynamic equilibrium, peaking the ionic conductivity at ~60 mS/cm. Concurrently, the uniform reinforcement of the water-mediated H-bonding network elevates the tensile strength to ~6.5 MPa. In another application, Santiago-Alonso et al. [120] utilized BDS to analyze Poly(vinylidene fluoride) (PVdF)-based ionogels, revealing that the addition of lithium salts reduces the polymer crystallinity by weakening the electrostatic interactions between the IL anions and cations. Under polymer confinement, polymorphic transitions are suppressed, and the disordered conducting phase is stabilized, enabling the ionic conductivity under specific conditions to reach twice that of the bulk liquid IL. VFT fitting of the dielectric relaxation profiles revealed lower activation energies under confinement, indicating that nanostructural restriction suppresses ion aggregation to achieve a decoupled yet simultaneous enhancement of both ion transport and structural stability (Figure 8a–c). To resolve overlapping responses in multi-component systems, Darwish et al. [121] employed BDS to investigate sodium alginate-based conductive hydrogels, resolving superimposed spectra arising from electrode polarization, charge transport, and molecular relaxations over a wide frequency sweep. By subtracting the DC-conductivity contributions, they successfully isolated the masked dielectric relaxation processes, demonstrating the efficacy of BDS in elucidating multi-mechanism competition within highly conductive, multi-phase systems.

However, as a macroscopic averaging technique, BDS cannot directly provide real-space trajectories of ion migration pathways, localized H-bond cleavage/recombination events, or interfacial chemical states. Moreover, electrode polarization in highly conductive systems often masks key molecular relaxation peaks. Therefore, establishing a comprehensive structure–property relationship requires multiscale, multi-method cross-validation. This approach combines macroscopic BDS with ion self-diffusion coefficients from SSNMR, ion association states from Raman spectroscopy, and localized H-bond lifetimes from MD simulations to span from bulk conductivity to microscopic ion hopping and segment dynamics [122].

#### 6.1.2. Pulsed-Field Gradient Nuclear Magnetic Resonance (PFG-NMR)

PFG-NMR enables the in situ, electrode-free characterization of ion diffusion at the molecular scale, eliminating the interfacial polarization and electrochemical side reactions associated with conventional electrode-based measurements. This makes it exceptionally suitable for highly viscous, strongly H-bonded systems such as cellulose ionogels. By measuring the self-diffusion coefficients (*D*) of anions and cations, PFG-NMR determines ion transference numbers and tracks the localized diffusion of characteristic nuclei (e.g., ^7^Li or ^19^F), providing crucial experimental evidence to establish quantitative links between network microstructure and macroscopic ion transport properties.

In their study on acrylic-based ionic gels, Igbaroola et al. [79] demonstrated that Li^+^ exhibits superior diffusion efficiency within the polymer network compared to free-state ILs, providing a critical kinetic rationale for its application as a high-performance solid electrolyte. Beyond measuring diffusion coefficients, PFG-NMR resolves the distribution of ion dissociation states, coordination dynamics, and localized transport heterogeneity under nanostructural confinement. This enables the establishment of a cross-scale quantitative correlation pathway ranging from interfacial H-bond reconstruction and segment-coordination environments to ion diffusion anisotropy and macroscopic ion transference numbers. Methodologically, Duncan et al. [123] employed PFG-NMR to characterize ion diffusion in Cu^2+^-doped cellulose nanofiber solid electrolytes. By tracking the ^7^Li and ^19^F signals and applying the classical Stejskal–Tanner equation, they calculated the self-diffusion coefficients of Li^+^ and TFSI^−^, obtaining an ion diffusion coefficient of approximately 5 × 10^−12^ m^2^/s at 45 °C. This study highlights the unique capacity of PFG-NMR to elucidate interactions between migrating ions and the polymer matrix, distinguish diffusion behaviors across heterogeneous coordination environments, and provide molecular-level insights into transport mechanisms within multi-tiered electrolytes (Figure 8d).

Nevertheless, PFG-NMR has inherent limitations in resolving fast chemical exchange processes, short-range correlated motions, and the fine morphology of sub-nanometer confined channels, making it difficult to decouple the limiting effects of physical entanglement from those of chemical coordination on diffusion. Future studies should integrate SAXS for the real-time tracking of nanostructural evolution, utilize PFG-NMR to simultaneously map self-diffusion coefficients and transference numbers, and perform rheological measurements to track network elasticity. Integrating these three approaches is essential to elucidate the dynamic coupling and decoupling mechanisms among structural rearrangement, ion transport, and bulk mechanical responses.

#### 6.1.3. Electrochemical Impedance Spectroscopy (EIS)

EIS is a classical characterization technique for resolving the electrochemical responses and charge migration kinetics of various ion-conducting materials [124]. When applied to ionogels, EIS captures key parameters, including bulk resistance, double-layer capacitance, charge-transfer resistance, and reaction kinetics [125]. By monitoring temperature- or composition-dependent changes in bulk ionic conductivity, EIS indirectly reflects both the H-bonding interactions between cellulose functional groups and the IL phase, and the regulatory effects of the three-dimensional fibrous pore network on overall ion mobility. Furthermore, EIS enables researchers to evaluate how structural fillers or network engineering optimize the ion-conduction pathways, exposing the mechanisms by which ion aggregation and dissociation dictate electrochemical performance.

Yu et al. [126] employed EIS to determine the bulk resistance of cellulose ionogels and calculate their ionic conductivity, quantitatively evaluating the impact of carboxymethylation and Na^+^ doping on ion transport. Their EIS analysis demonstrated that carboxymethylation expands the available ion transport pathways, yielding a significantly higher conductivity for carboxymethylated bacterial cellulose-based ionogels (24.8 mS/cm) compared to pristine bacterial cellulose ionogels (13.8 mS/cm). Although the subsequent addition of Na^+^ slightly decreased conductivity due to increased viscosity, it synergistically enhanced the overall thermoelectric power factor, providing critical electrical parameters for structure–property relationship studies (Figure 8e). Similarly, Li et al. [127] utilized EIS as the primary characterization tool to quantify the ionic conductivity of a crystallization-assisted, dual-ion complexed cellulose ionogel. Through Nyquist plot analysis, they attributed the gel’s superior conductivity to crystallization-induced network densification and a highly ordered molecular pathway formed through dual-ion coordination. Temperature-dependent EIS measurements further confirmed the material’s excellent environmental adaptability, maintaining stable performance across a wide temperature sweep from −40 °C to 80 °C. In another study, Li et al. [128] evaluated the ionic conductivity of cellulose-based ionogels by fitting equivalent circuits (R_1_ + CPE_1_) to experimental Nyquist plots, calculating the conductivity via:

*σ*_i_ = l/(R_b_ ⋅A)

where l is the sample thickness, A is the contact area, and R_b_ is the bulk resistance. The results demonstrated that the structurally optimized, moderate-molecular-weight cellulose ionogel forms a dense network with highly efficient ion channels, achieving a conductivity of 69 mS/cm—significantly higher than that of its low-molecular-weight counterpart (33 mS/cm), thereby validating the regulatory role of macromolecular structure on macroscopic charge transport.

While EIS excels at resolving macroscopic charge transfer and boundary resistance, its spatial resolution is limited, making it difficult to distinguish between localized structural domains and distinct relaxation behaviors. Future studies must integrate in situ scattering techniques with MD simulations to establish cross-scale correlations between interfacial double-layer capacitance and atomic-scale ion density distributions, thereby mapping structure-transport relationships from equivalent circuits to atomic configurations.

### 6.2. Dynamic Analysis of Mechanical Properties and Transport-Mechanics Synergy

To overcome the classical strength-conductivity trade-off, cellulose ionogels rely on three structural design strategies:

Biphasic Hard/Soft Microphase Separation: Rigid polymer-rich domains (high H-bond density) form a continuous structural skeleton to provide mechanical strength (*E*, *σ_t_*), while interconnected solvent/IL-rich domains form continuous channels for rapid vehicular/hopping ion transport [129].

Dynamic Sacrificial Physical Networks: Reversible non-covalent interactions (H-bonds, ion-coordination) act as sacrificial bonds that fracture under deformation to dissipate strain energy (yielding high toughness and low fatigue hysteresis) and rapidly reform post-unloading, while ion hopping continues independently through the matrix [130].

Water/Solvent-Mediated Interfacial Optimization: Controlled solvent addition plasticizes H-bonded networks to enhance strain while simultaneously promoting ion-pair dissociation (CIP→SIP), achieving simultaneous increases in toughness and conductivity [131].

To provide quantitative design guidance, Table 3 compiles representative cellulose ionogels, illustrating how composition, IL species, water content, and temperature govern transport-mechanical synergy.

#### 6.2.1. In Situ Stretching and Cyclic Loading

In situ stretching and cyclic loading tests serve as pivotal experimental approaches for elucidating the structure–property relationships of cellulose ionogels under mechanical deformation. Real-time monitoring of stress–strain curves enables researchers to differentiate between the rigid support provided by covalently cross-linked backbones and the energy dissipation/reinforcement effects associated with physical sacrificial networks (such as H-bonds, ion coordination, and chain entanglements) during stretching. Cyclic loading-unloading curves quantify the dynamic fracture and reversible recovery behaviors of multiple non-covalent interactions, reflecting the network’s elastic recovery, fatigue resistance, and interphase load-transfer efficiency. Furthermore, performing in situ stretching under variable temperature conditions allows for the systematic assessment of structural stability and mechanical retention mechanisms across different thermal environments [133]. This integrated methodology facilitates an understanding of how molecular chain alignment, phase-separation kinetics, and multiscale network restructuring govern macroscopic mechanical properties, providing a critical experimental foundation to correlate network topology with bulk mechanics from the perspective of microscale interactions.

**Figure 9:**
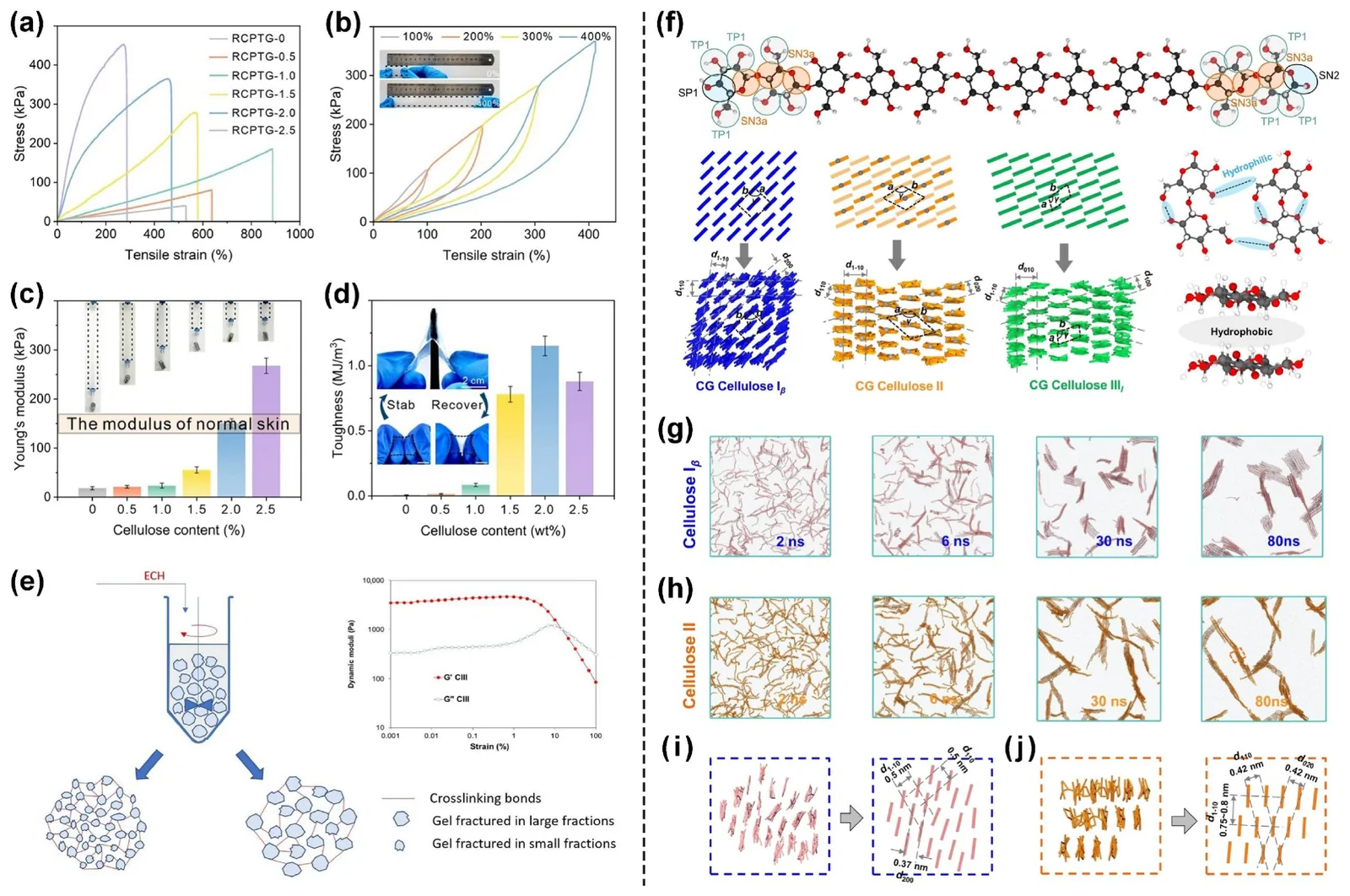
(**a**) Tensile stress–strain curve of RCPTGs. (**b**) Stretching cycle curves of RCPTGs. (**c**) Young’s modulus of RCPTGs. (**d**) Comparison of toughness of RCPTGs. Reprinted with permission from [132]. Copyright 2024, Springer Nature. (**e**) A model for the hydrogel synthesis and the amplitude sweep test results. Reprinted with permission from [136]. Copyright 2022, MDPI. (**f**) Martini 3 modeling of cellulose. A series of snapshots of the (**g**) cellulose I*_β_* and (**h**) cellulose II regeneration process. Sectional views of the regenerated (**i**) cellulose I*_β_* and (**j**) cellulose II crystallities and the corresponding simplified stacking schematic. Reprinted with permission from [138]. Copyright 2023, Elsevier.

Feng et al. [134] conducted cyclic loading-unloading tensile tests to investigate the mechanical deformation and energy dissipation mechanisms of ionogels, demonstrating the design advantages of a dual-crosslinked, semi-interpenetrating network. Their study revealed two key insights: first, the reversible H-bonds within the cellulose matrix can act as physical sacrificial bonds that dissipate strain energy during stretching and rapidly recover post-unloading—enabling excellent fatigue resistance with a hysteresis rate of only 21.7% after 500 continuous cycles; second, it established direct correlations between macroscopic mechanics (stress–strain profiles, hysteresis rates) and microstructural parameters (crosslink density, pore architecture), validating a structure–property pipeline linking microstructure-mechanical behavior -functional stability. Similarly, Du et al. [132] investigated the impact of microphase-separated domains on ionogel mechanics and energy dissipation. Their results showed that a regenerated cellulose/PHEMA triboelectric gel (RCPTG) exhibited no significant hysteresis under increasing strain. This was attributed to the sacrificial dissipation of reversible H-bonds in the rigid phase combined with the moderate crosslinking of flexible networks in the soft phase, proving that alternating hard-soft phases enable efficient interfacial load transfer and providing direct experimental evidence of the relationship among phase separation, mechanical toughness, and sensing stability (Figure 9a–d).

Although in situ stretching and cyclic loading tests can identify the macroscopic sites and temporal thresholds of H-bond reconstruction, their limited signal resolution prevents the identification of overlapping vibrational peaks, localized electronic structures, or transient, short-lived coordination species. Future research should integrate spectroscopic detection (e.g., in situ AFM-IR or Raman) with molecular simulations to analyze interfacial behavior and phase separation across multiple length scales, thereby advancing the study of dynamic structure–property relationships in cellulose ionogels.

#### 6.2.2. Dynamic Mechanical Analysis (DMA)

DMA applies alternating oscillatory forces to materials and monitors changes in the *G*′ and *G*″ to characterize viscoelastic properties. When applied to cellulose ionogels, DMA enables differentiation between the individual contributions of physical and chemical crosslinks, while offering visual evidence of solvent-cellulose interfacial interactions, H-bond dynamics, and polymer chain segment relaxation.

For example, Elf et al. [135] measured the variation in *G*’ with temperature using DMA, finding that the ring-opening modification of diol cellulose significantly reduces the material’s stiffness above the *T_g_* while having minimal effect at room temperature. This demonstrated how molecular-scale structural modifications can regulate macroscopic thermomechanical properties, illustrating that DMA establishes quantitative relationships between microscopic molecular motions (e.g., segment mobility) and macroscopic mechanical properties (e.g., modulus and toughness). Additionally, Ciolacu et al. [136] conducted frequency sweeps on chemically cross-linked hydrogels prepared from different cellulose polymorphs (I, II, III). They observed that all samples exhibited *G*′ values consistently higher than *G*″ without any crossover points over the tested frequency range, confirming the formation of highly stable, covalently cross-linked networks. They further noted that cellulose II-derived hydrogels exhibited lower crosslinking densities and higher swelling ratios due to their higher gel-stage strength and larger shear fracture fragments (Figure 9e).

Because conventional DMA struggles to directly distinguish the coexistence and individual contribution ratios of concurrent dynamic interactions (such as physical vs. chemical crosslinks), future investigations should integrate in situ vibrational spectroscopy (for real-time monitoring of H-bond and covalent-bond evolution), diffusion spectroscopy, and theoretical models (e.g., coupling reaction kinetics with network theory) to establish cross-scale correlations spanning from molecular motion to macroscopic viscoelastic behavior.

#### 6.2.3. Theoretical Mechanical Modeling

Within the framework of theoretical mechanics, integrating FEA with MD simulations provides a multiscale paradigm for investigating stress distribution and fracture mechanics, spanning from atomistic bonds to macroscopic networks. FEA, by constructing RVE models, characterizes the elastic modulus and stress-transfer pathways within cellulose fiber networks, while molecular mechanics models elucidate at the atomistic level how H-bond reorganization, chain segment coordination, and solvent-cellulose interactions govern the gel’s mechanical properties.

For example, Xie et al. [137] developed a multiscale finite element model encompassing composite domains, single fibers, and fiber networks to investigate the tensile properties of cellulose membranes systematically. They found that the longitudinal elastic modulus *E_LD_* of cellulose microfibers increases significantly with rising cellulose volume fraction. In contrast, the transverse elastic modulus *E_TD_* is governed primarily by the bonding interactions between hemicellulose and lignin. Their simulations showed that stress within the fiber network is concentrated predominantly in the H-bonded regions at fiber junctions. Additionally, Pang et al. [138] employed Martini 3 CG MD simulations combined with reverse mapping techniques to resolve the structural evolution of single-molecule-layer sheets during cellulose regeneration, showing that hydrophobic interactions and H-bond stacking drive the formation of type II cellulose crystals, providing atomic-scale insights into segmental rearrangement and stress distribution within the gel network (Figure 9f–j).

However, single-scale mechanical models (such as those based solely on FEA or CG simulations) struggle to simultaneously account for both the macroscopic topological structure of large-scale fiber networks and the dynamic, molecular-scale cleavage-reconstruction of H-bonds. Furthermore, CG models exhibit limitations in predicting the thermodynamic stability of specific cellulose crystal polymorphs. Future research trends must focus on integrating finite element simulations and CG MD with in situ characterization techniques like AFM and XRD, utilizing multiscale parameter inversion and machine learning-assisted force field optimization to achieve high-precision predictions of stress–strain behavior and ion-transport coupling mechanisms under real-world operating conditions [139].

## 7. Challenges and Prospects

Cellulose ionogels represent a highly promising class of sustainable functional materials, combining exceptional biocompatibility with tunable mechanical resilience and superior ionic conductivity. Decoding their intricate multiscale structure–property relationships demands a synergistic paradigm coupling real-time in situ/operando characterization with advanced theoretical modeling. Despite significant recent strides, critical bottlenecks remain in cross-scale data integration, high-fidelity modeling, and industrial scalability. Future research should prioritize the following directions:

### 7.1. Bridging Spatiotemporal Scales via Physics-Informed Machine Learning (PIML) and Machine Learning Potentials (MLPs)

A fundamental challenge in modeling multi-component cellulose ionogels (cellulose, ILs, water, co-solvents) is the stark trade-off between quantum-chemical accuracy and classical MD spatiotemporal reach. Standard DFT calculations are restricted to small atomic clusters (<500 atoms) at 0 K, whereas classical MD with empirical force fields often lacks explicit electronic polarization and fixed-charge transfer descriptions.

To bridge DFT accuracy with MD-accessible length/time scales (nanoseconds/nanometers), MLPs—such as neural network potentials (e.g., DeepMD, ANI, SchNet) trained on extensive DFT-calculated potential energy surfaces (10^4^–10^6^ atomic configurations)—are playing a transformative role. MLPs execute O(N) linear scaling, enabling quantum-accurate MD simulations of dynamic H-bond breaking/reforming, ion-pair dissociation kinetics, and local solvation shell restructuring over extended timescales. Furthermore, PIML incorporates foundational physical laws (e.g., mass/charge conservation, thermodynamic equilibrium bounds, and Arrhenius/VFT transport constraints) directly into loss functions. PIML enables bidirectional parameter transfer between atomistic MD trajectories, mesoscopic SAXS correlation lengths, and continuum FEA stress distributions, establishing closed-loop cross-scale parameter inversion.

### 7.2. Concrete Workflows for AI-Driven Inverse Material Design

While AI-driven material discovery is promising, current efforts often remain purely conceptual. To achieve true predictive, targeted reverse design of high-performance cellulose ionogels, we outline a concrete, data-driven workflow encompassing specific inputs, descriptors, machine learning architectures, and validation protocols:

Model Inputs and Descriptors:

Molecular & Chemical Descriptors: Topological molecular fingerprints (Morgan/MACCS keys) and SMILES representations of IL cations/anions; Kamlet–Taft parameters (*α*, *β*, *π*^∗^); COSMO-RS *σ*-profiles and activity coefficients; solvent molar volume and dielectric constants.

Structural & Processing Descriptors: Cellulose degree of polymerization (*DP*), Crystallinity Index (*CI*), H-bond density, network mesh size (*ξ*), microphase domain spacing (*d*), cellulose content (wt%), water/additive ratio (wt%), dissolution temperature, shear rate, and coagulation bath composition.

Predicted Target Outputs:

Room-temperature and low-temperature ionic conductivity (*σ*_i_); tensile strength (*σ*_t_); Young’s modulus (*E*); fracture toughness (*U*_T_); cycling fatigue hysteresis rate; thermal decomposition temperature (*T*_d_); and electrochemical stability window (*EVW*).

Dataset Sizes and Repositories: Current training relies on small-scale literature datasets (10^2^–10^3^ experimental entries) and high-throughput virtual screening libraries (e.g., COSMO-RS libraries with >10^5^ IL candidates). Developing open-access, standardized benchmark databases containing multi-component thermodynamic and transport parameters is urgently required.

Machine Learning Architectures: Graph Neural Networks (e.g., SchNet, MEGNet) to learn structural representations of entangled cellulose–IL networks; XGBoost/Random Forest for rapid property prediction; and Generative Adversarial Networks or Variational Autoencoders for generating candidate IL/solvent formulations targeting pre-specified performance thresholds.

Validation Strategies: Nested *k*-fold cross-validation, out-of-distribution generalization testing across diverse cellulose sources (microcrystalline, bacterial, wood pulp), and automated closed-loop robotic synthesis coupled with high-throughput EIS and tensile testing for experimental verification.

### 7.3. Industrial Translation: Scalability, Long-Term Stability, Recyclability, and Reproducibility

Before cellulose ionogels can achieve commercial translation in wearable electronics and energy storage, four major engineering and manufacturing bottlenecks must be critically addressed:

Manufacturing Scalability: Lab-scale fabrication predominantly relies on batch dissolution and manual casting. Transitioning to industrial-scale manufacturing demands continuous roll-to-roll processing, automated solvent exchange, and rapid, uniform coagulation kinetics over large surface areas without structural warping or micro-bubble entrapment.

Long-Term Environmental Stability: Cellulose ionogels contain dynamic, hygroscopic networks that are sensitive to ambient relative humidity and temperature fluctuations. In dry environments, solvent evaporation leads to matrix dehydration, stiffness, and loss of ionic conductivity; under extreme humidity, excessive water absorption causes network swelling and plasticization failure. Waterproof encapsulation strategies, non-volatile IL formulations, or chemical crosslinking that maintains dimensional stability without sacrificing foldability are essential.

Recyclability and Closed-Loop Circularity: Unlike non-recyclable petroleum-based synthetic elastomers, the physical non-covalent network of cellulose ionogels offers intrinsic reprocessability. Immersing used ionogels in selective solvents (e.g., water or ethanol) disrupts interchain H-bonds, allowing complete disassembly into redispersible nanocellulose suspensions and reusable ILs. Developing low-energy, green recovery protocols for expensive ILs and active functional additives (e.g., AgNWs, MXenes) is critical for a circular materials economy.

Manufacturing Reproducibility: Natural cellulose precursors exhibit inherent batch-to-batch variations in molecular weight distribution, hemicellulose/lignin content, and *DP*. Establishing standardized feedstock refining protocols and quality-control benchmarks is mandatory to ensure reproducible viscoelastic gelation kinetics and uniform mechanical/electrical properties across manufacturing batches.

### 7.4. Critical Role of Mesoscopic Phase Separation

A key insight emerging from recent reports is that mesoscopic phase separation serves as the central structural conduit for decoupling traditionally conflicting properties:

Influence on Ionic Conductivity: Bicontinuous microphase separation establishes interconnected, solvent-rich low-viscosity channels that enable rapid vehicular and hopping ion transport, yielding high conductivity (*σ*_i_ > 10 mS cm^−1^).

Influence on Mechanical Toughness: Dense, polymer-rich domains assemble into a rigid H-bonded structural framework (*E* > 10 MPa). Under stress, reversible physical crosslinks within this hard phase act as sacrificial bonds, fracturing to dissipate viscoelastic strain energy while the flexible soft phase accommodates deformation, yielding high fracture toughness (*U*_T_) and fatigue resistance.

Influence on Thermal Stability: Confinement of IL molecules within dense nanocellulose domains elevates activation energy barriers for thermal volatilization, extending thermal endurance beyond 120 °C.

## 8. Conclusions

The structural complexity and versatile macroscopic performance of cellulose ionogels are fundamentally rooted in the dynamic, hierarchical coupling of their molecular, mesoscopic, and macroscopic architectures. Unraveling these intricate structure–property relationships necessitates a multidimensional, cross-disciplinary approach. Multiscale in situ characterization provides real-time, physical snapshots of structural evolution during processing and service, while theoretical simulations decode atomistic solvation and thermodynamic driving forces.

Rather than offering a simple descriptive summary, this review synthesizes recent application advancements and formulates six concrete, actionable design principles for engineering next-generation cellulose ionogels:

Interaction Energy Tuning Principle: Solvation capacity must be optimized by selecting IL anions whose H-bond basicity (*β*) and molecular volume strike a delicate balance: strong enough to disrupt native interchain O_3_H…O_5_ and O_6_H…O_3_′ H-bonds for molecular dispersion, yet mild enough to prevent nucleophilic degradation of glycosidic backbones.

Biphasic Microphase Separation Principle: Designing bicontinuous hard/soft microdomains effectively resolves the classical strength-conductivity trade-off. Hard, polymer-dense regions provide structural rigidity (*E*, *σ*_t_), while soft, solvent-rich channels serve as low-barrier highways for rapid vehicular/hopping ion migration (*σ*_i_).

Plasticization–Dissociation Balance Principle: Incorporating controlled moisture or high-dielectric co-solvents (~20–30 wt%) plasticizes rigid H-bonded networks to increase fracture strain while simultaneously promoting CIP to SIP transitions, maximizing charge carrier density (*n*_j_).

Polymorphic and Crystallinity Control Principle: Regulating the Cellulose I to Cellulose II polymorphic transition during coagulation stabilizes disordered, highly conducting amorphized phases while retaining sufficient crystalline domains to preserve dimensional stability across wide temperature windows (−40 °C to 120 °C).

Dynamic Sacrificial Crosslinking Principle: Combining reversible physical junctions (H-bonds, ion coordination) with permanent covalent/entangled networks creates sacrificial energy dissipation pathways that fracture under strain and rapidly reform post-unloading, ensuring high toughness and low cyclic fatigue hysteresis.

Confinement and Activation Energy Principle: Utilizing nanostructural confinement lowers activation energy barriers (*E*_a_) for dielectric relaxation and ion hopping, suppressing ion aggregation and maintaining superior charge transport in extreme operating environments.

Ongoing innovations in operando multi-field diagnostics, hybrid Quantum Mechanics/Molecular Mechanics modeling, MLPs, and AI-driven inverse design workflows will profoundly deepen our understanding of cellulosic ionic soft matter. Ultimately, this multiscale synergistic paradigm establishes a robust theoretical foundation and technical blueprint for the predictive design, circular manufacturing, and commercial translation of sustainable cellulose ionogels in next-generation flexible electronics and energy storage technologies.

## Figures and Tables

**Table 1:** Comprehensive comparison of computational and theoretical modeling methodologies applied to cellulose–IL systems.

Method	Applicable Length/Time Scale	Predictive Capability & Key Outputs	Computational Cost	Core Assumptions & Limitations	Application in Ionogel Research	Ref.
COSMO-RS	Bulk phase/Thermodynamic equilibrium	Solvation energy (Δ*G_solv_*), activity coefficients (lnγ), excess enthalpy (*H^E^*), high-throughput IL screening	Very Low (Seconds to minutes per system)	Assumes thermodynamic equilibrium; ignores dynamic conformational evolution, steric hindrance, and explicit oxygen H-bonding geometry	Screening optimal IL anions/cations, solvent miscibility, binary/terinary phase behavior	[54]
DFT	Sub-nanometer (<2 nm)/Static ground states	Electronic structures, charge transfer, H-bond binding energies, core-level binding energy shifts	High to Prohibitive (Hours to days per cluster)	Restricted to small molecular clusters (<200–500 atoms); static calculations at 0 K; neglects long-range cooperative dynamics and entropic effects	Anion–hydroxyl binding strength, ion-pair dissociation, antisolvent-induced H-bond cleavage	[44]
MD	2–50 nm/1–500 ns	Atomistic diffusion trajectories, Radial Distribution Functions (RDFs), H-bond lifetimes, ion hopping kinetics	Moderate to High (Days to weeks)	Dependent on empirical force field parameterization; cannot capture chemical bond breaking/forming or explicit electronic polarization	Chain unwinding kinetics, solvation shell restructuring, ion transport pathways	[55]
CG-MD	10–500 nm/0.1–10 µs	Mesoscopic phase separation, polymer entanglement, self-assembly kinetics, topological network morphology	Moderate (Hours to days)	Coarsening reduces atomic-scale chemical fidelity; struggles to predict specific crystal polymorph stability (e.g., Cellulose I vs. II)	Sol-to-gel transition, microphase domain formation, network densification	[56]
FEA	Micrometers to millimeters/Continuum	Representative Volume Element (RVE) stress distribution, bulk elastic modulus, fracture energy dissipation	Low to Moderate (Minutes to hours)	Treats materials as continuous media; neglects molecular H-bond breaking/reforming and nanoscale thermal fluctuations	Fiber network mechanics, anisotropic strain distribution, structural mechanics	[39]
MLP	5–100 nm/1–100 ns	Quantum-accurate atomic trajectories, dynamic reactive pathways at near-classical MD computational speeds	Moderate (Heavy initial DFT training, fast inference)	Requires extensive high-level DFT training datasets; limited transferability beyond trained chemical domains	Bridging DFT electronic accuracy with MD-accessible length/time scales for complex multi-component gels	[57]

**Table 2:** Comparison of major in situ and operando characterization techniques for cellulose ionogels.

Technique	Temporal/Spatial Resolution	Measurable Parameters & Information	Principal Limitations & Technical Challenges	Ref.
In Situ FTIR/2D-COS	~ms to s/Bulk (~µm)	Sequential H-bond cleavage/reconstruction, functional group coordination, sol-to-gel kinetics.	Severe spectral peak overlap; bulk averaging without 3D spatial resolution.	[71]
In Situ Raman	~s/~1 µm	Ring-stacking, homopolar bond vibrations, anion preferential solvation shifts.	Fluorescence interference; lower sensitivity to polar OH groups than FTIR.	[72]
APXPS/XPS	~s to min/Surface (<10 nm)	Core-level binding energy shifts, charge transfer, interfacial electronic states.	High surface sensitivity; X-ray beam damage under prolonged exposure; strict vacuum/humidity constraints.	[73]
In Situ SANS	~s to min/1–100 nm	SLD contrast, quantitative IL anion binding ratio, bound ion solvation sheath, effective chain charging, contrast-variation chain conformations.	Requires expensive/complex isotopic deuteration; requires reactor/spallation neutron beamtime access; incoherent background noise.	[74]
In Situ SAXS	~ms to s/1–100 nm	Network mesh size (*ξ*), domain spacing (*q_max_*), microphase separation kinetics.	Operates on electron density contrast; cannot distinguish specific chemical bond types.	[75]
In Situ WAXS/XRD	~ms to s/0.1–2 nm	Atomic chain packing, crystal polymorph transitions (Cellulose I to II), crystallinity index (*CI*).	Insensitive to amorphous matrix domains and transient non-crystalline H-bonding networks.	[76]
In Situ Rheology	~s/Bulk	Viscoelastic moduli (*G*′, *G*″), gel point (*T_gel_*), crosslink density kinetics.	Bulk averaging technique; cannot identify chemical nature of specific crosslink sites.	[77]
AFM PF-QNM	~ms per curve/~nm	Local Young’s modulus, surface deformation, nanoscale tip-sample adhesion mapping.	Surface-bound measurement; prone to tip contamination and confinement artifacts.	[78]
PFG-NMR	~ms/Bulk	Self-diffusion coefficients (*D*), ion transference numbers (*T_+_*), transport anisotropy.	Cannot resolve fast chemical exchange or sub-nanometer confinement morphological details.	[79]
BDS	~µs to s/Bulk	Dipole polarization relaxations, charge transport kinetics, dielectric relaxation activation energy.	Electrode polarization masks low-frequency molecular relaxation peaks in high-conductivity gels.	[80]

**Table 3:** Composition, processing conditions, and quantitative transport-mechanical metrics in representative cellulose ionogels.

Cellulose Source/Content (wt%)	IL Type	Solvent/Co-Solvent	Water Content (wt%)	Operating Temp (°C)	Ionic Conductivity (mS cm^−1^)	Tensile Strength (MPa)	Elastic Modulus (MPa)	Fracture Strain (%)/Toughness	Ref.
Wood Powder Cellulose (4.0 wt%)	[EMIM][BF_4_]	[BMIM]Cl	~0	25	14.3	3.5	12.8	320%	[14]
Patterned Cellulose (7.1 wt%)	[BMIM][Cl]	Acetonitrile	~0	25	12.9	3.7	5.5	280%	[15]
Cotton Cellulose (2.0 wt%)	[BzMIM][MeOAc]	-	~0	80	8.5	0.4	0.9	110%	[18]
Nanocrystalline Cellulose (8.0 wt%)	[BMIM][Cl]	DMF	~0	25	18.2	11.5	35.0	470%	[38]
Microcrystalline Cellulose (3.0 wt%)	[BMIM][OAc]	DMSO	~0	25	12.5	0.8	1.2	150%	[49]
Wood Pulp Cellulose (5.0 wt%)	[EMIM][DEP]	Water	~30	25	60.0	6.5	18.5	320%	[119]
Bacterial Cellulose (4.0 wt%)	[EMIM][OAc]	Water	~10	25	24.8	2.1	5.4	210%	[126]
Carboxymethyl Cellulose (5.0 wt%)	[EMIM][TFSI]	Water	~20	25	28.5	5.1	14.2	390%	[128]
Regenerated Cellulose (4.0 wt%)	[BMIM][TFSI]	PHEMA	~0	25	15.4	4.2	8.6	520%	[132]

## Data Availability

No new data were created or analyzed in this study. Data sharing is not applicable to this article.

## Abbreviations

2D-COS	Two-Dimensional Correlation Spectroscopy
AFM	Atomic Force Microscopy
APXPS	Ambient-Pressure X-ray Photoelectron Spectroscopy
BDS	Broadband Dielectric Spectroscopy
CG	Coarse-Grained
CG-MD	Coarse-Grained Molecular Dynamics
CI	Crystallinity Index
CIP	Contact Ion Pair
CNF	Cellulose Nanofiber
COSMO-RS	Conductor-like Screening Model for Real Solvents
Cryo-SEM	Cryogenic Scanning Electron Microscopy
Cryo-TEM	Cryogenic Transmission Electron Microscopy
DFT	Density Functional Theory
DMA	Dynamic Mechanical Analysis
DMSO	Dimethyl Sulfoxide
DP	Degree of Polymerization
EDS	Energy-Dispersive X-ray Spectroscopy
EIS	Electrochemical Impedance Spectroscopy
FEA	Finite Element Analysis
FTIR	Fourier-Transform Infrared Spectroscopy
IL	Ionic Liquid
MD	Molecular Dynamics
MLP	Machine Learning Potential
NMR	Nuclear Magnetic Resonance
PF-QNM	PeakForce Quantitative Nanomechanical Mapping
PFG-NMR	Pulsed-Field Gradient Nuclear Magnetic Resonance
PIML	Physics-Informed Machine Learning
PLM	Polarized Light Microscopy
PVA	Poly(vinyl alcohol)
PVdF	Poly(vinylidene fluoride)
RDF	Radial Distribution Function
RVE	Representative Volume Element
SANS	Small-Angle Neutron Scattering
SAXS	Small-Angle X-ray Scattering
SEM	Scanning Electron Microscopy
SIP	Solvent-Separated Ion Pair
SSNMR	Solid-State Nuclear Magnetic Resonance
TEM	Transmission Electron Microscopy
VFT	Vogel–Fulcher–Tammann
WAXS	Wide-Angle X-ray Scattering
XPS	X-ray Photoelectron Spectroscopy
XRD	X-ray Diffraction
